# Multimodal Imaging and Analysis of the Neuroanatomical Organization of the Primary Olfactory Inputs in the Brownbanded Bamboo Shark, *Chiloscyllium punctatum*

**DOI:** 10.3389/fnana.2020.560534

**Published:** 2020-11-26

**Authors:** Victoria Camilieri-Asch, Harrison T. Caddy, Alysia Hubbard, Paul Rigby, Barry Doyle, Jeremy A. Shaw, Andrew Mehnert, Julian C. Partridge, Kara E. Yopak, Shaun P. Collin

**Affiliations:** ^1^School of Biological Sciences, The University of Western Australia, Perth, WA, Australia; ^2^The Neuroecology Group, UWA Oceans Institute, The University of Western Australia, Perth, WA, Australia; ^3^Vascular Engineering Laboratory, Centre for Medical Research, Harry Perkins Institute of Medical Research, The University of Western Australia, Perth, WA, Australia; ^4^School of Engineering, The University of Western Australia, Perth, WA, Australia; ^5^Centre for Microscopy, Characterisation and Analysis, The University of Western Australia, Perth, WA, Australia; ^6^Australian Research Council Centre for Personalised Therapeutics Technologies, Perth, WA, Australia; ^7^BHF Centre for Cardiovascular Science, The University of Edinburgh, Edinburgh, United Kingdom; ^8^National Imaging Facility, Brisbane, QLD, Australia; ^9^Department of Biology and Marine Biology, Center for Marine Science, University of North Carolina Wilmington, Wilmington, NC, United States; ^10^School of Life Sciences, La Trobe University, Melbourne, VIC, Australia

**Keywords:** olfactory pathway, elasmobranch, diceCT, LM, SEM, TEM, IHC, 3D simulations

## Abstract

There is currently a limited understanding of the morphological and functional organization of the olfactory system in cartilaginous fishes, particularly when compared to bony fishes and terrestrial vertebrates. In this fish group, there is a clear paucity of information on the characterization, density, and distribution of olfactory receptor neurons (ORNs) within the sensory olfactory epithelium lining the paired olfactory rosettes, and their functional implications with respect to the hydrodynamics of incurrent water flow into the nares. This imaging study examines the brownbanded bamboo shark *Chiloscyllium punctatum* (Elasmobranchii) and combines immunohistochemical labeling using antisera raised against five G-protein α-subunits (Gα_s/olf_, Gα_q/__11__/__14_, Gα_i–__1__/__2__/__3_, Gα_i–__3_, Gα_*o*_) with light and electron microscopy, to characterize the morphological ORN types present. Three main ORNs (“long”, “microvillous” and “crypt-like”) are confirmed and up to three additional microvilli-bearing types are also described; “Kappe-like” (potential or homologous “Kappe” as in teleosts), “pear-shaped” and “teardrop-shaped” cells. These morphotypes will need to be confirmed molecularly in the future. Using X-ray diffusible iodine-based contrast-enhanced computed tomography (diceCT), high-resolution scans of the olfactory rosettes, olfactory bulbs (OBs), peduncles, and telencephalon reveal a lateral segregation of primary olfactory inputs within the OBs, with distinct medial and lateral clusters of glomeruli, suggesting a potential somatotopic organization. However, most ORN morphotypes are found to be ubiquitously distributed within the medial and lateral regions of the olfactory rosette, with at least three microvilli-bearing ORNs labeled with anti-Gα_*o*_ found in significantly higher densities in lateral lamellae [in lateral lamellae] and on the anterior portion of lamellae (facing the olfactory cavity). These microvilli-bearing ORN morphotypes (microvillous, “Kappe-like,” “pear-shaped,” and “teardrop-shaped”) are the most abundant across the olfactory rosette of this species, while ciliated ORNs are less common and crypt cells are rare. Spatial simulations of the fluid dynamics of the incurrent water flow into the nares and within the olfactory cavities indicate that the high densities of microvilli-bearing ORNs located within the lateral region of the rosette are important for sampling incoming odorants during swimming and may determine subsequent tracking behavior.

## Introduction

Extant cartilaginous fishes (Chondrichthyes), including chimaeras (Holocephali), sharks, skates, and rays (Elasmobranchii), represent a basal group of fishes in vertebrate phylogeny (Naylor et al., 2012; Yopak, 2012). Chondrichthyans constitute the earliest group of jawed fishes to exhibit a “basic” *bauplan* for brain organization, which is conserved in all later vertebrate taxa (Striedter, 2005), including bony fishes (Osteichthyes) and tetrapods (Amphibia, Reptilia, Aves, Mammalia). This brain *bauplan* includes the olfactory bulbs, telencephalon, diencephalon, mesencephalon, cerebellum, and medulla oblongata.

Olfaction has been thought to play an essential role in the ecology of cartilaginous fishes, mainly based on morphological traits, such as relatively large and elongated peripheral olfactory organs with highly folded epithelia, providing an increased sensory surface area, or relatively large olfactory bulbs, compared to bony fishes (Nieuwenhuys, 1966; Northcutt, 1977; Theisen et al., 1986; Zeiske et al., 1986; Lisney and Collin, 2006; Schluessel et al., 2008). However, it is still unclear whether the olfactory pathway of cartilaginous fishes is morphologically and functionally organized similarly to other jawed fishes, especially at the levels of signal detection and transduction, and the degree of convergence of olfactory inputs to the olfactory bulb (Collin et al., 2015; Yopak et al., 2015).

The peripheral olfactory system of many tetrapods (excluding birds and apes) can be divided into two anatomically distinct “sub-systems” or end organs (Northcutt, 1981). These include the main olfactory system (MOS) and the vomeronasal system (VNS), each of which possesses sensory epithelia populated with different olfactory receptor neurons (ORNs). Different ORNs express various sets of receptor molecules on their apical surfaces, coupled with defined G-protein α-subunits that mediate odorant signal transduction (Buck, 1996; Mombaerts, 2004). The olfactory sensory epithelium (or neuroepithelium) of the MOS principally contains ciliated ORNs, which express transmembrane receptor molecules from either the olfactory receptors (ORs) or the trace amine-associated receptor (TAAR) gene families, coupled to a Gα_*olf*_ subunit, and project to the main olfactory bulb (Jones and Reed, 1989; Buck and Axel, 1991; Liberles and Buck, 2006; Hashiguchi and Nishida, 2007). In contrast, the olfactory neuroepithelium of the VNS predominantly contains microvillous ORNs, which express vomeronasal receptors from two families of genes, V1Rs coupled to Gα_i_ subunits and V2Rs coupled to Gα_*o*_ subunits, and project to the accessory olfactory bulb (Shinohara et al., 1992; Berghard and Buck, 1996; Jia and Halpern, 1996). The olfactory receptors respond to a variety of ligands, where typically those expressed in the olfactory epithelium of the MOS respond to a range of odor molecules and detect overlapping ligand combinations, whereas VNS receptors have a strong affinity to specific ligands (Eisthen, 1997; Grus and Zhang, 2008; Spehr and Munger, 2009).

Unlike tetrapods, both cartilaginous and bony fishes possess a single, folded olfactory organ (olfactory rosette), covered (often only partially) by an olfactory neuroepithelium containing multiple ORN types (Broman, 1920; Eisthen, 1992). To date, five different ORN types have been characterized in teleosts (ciliated, microvillous, crypt, Kappe, and pear) (Zeiske et al., 1992; Morita et al., 1996; Hansen and Zeiske, 1998; Hansen and Finger, 2000; Hansen and Zielinski, 2005; Saraiva and Korsching, 2007; Heffern et al., 2018; Calvo-Ochoa and Byrd-Jacobs, 2019), two of which (ciliated and microvillous) appear to be homologous with the receptor classes and associated G-proteins reported in mammals (Hansen et al., 2003; Biechl et al., 2017). Crypt ORNs express an ancestral V1R receptor associated with Gα_i_ (Hansen et al., 2003; Oka et al., 2011), or Gα_*q*_, and Gα_*o*_ (Hansen et al., 2004), depending on the teleost species. Kappe ORNs express an unknown receptor type, but are associated with Gα_*o*_ (Ahuja et al., 2014), and pear ORNs express a new type of receptor (A2c) associated with Gα_*olf*_ (Wakisaka et al., 2017). To date, only two types of ORNs have been recognized in chondrichthyans (microvillous and crypt) (Theisen et al., 1986; Takami et al., 1994; Ferrando et al., 2006b, 2007, 2010, 2016, 2009; Schluessel et al., 2008; Theiss et al., 2009), which are associated with either Gα_*o*_ and/or Gα_i_. No studies to date have found ciliated, Kappe or pear ORNs in any species of cartilaginous fish.

Although several studies have focused on the morphology of the olfactory epithelium in both cartilaginous and bony fishes, none have examined the number, density, and distribution of ORNs in chondrichthyans. While a differential distribution of ORNs has been shown in some teleost species, including the common goldfish *Carassius auratus* (Hansen et al., 2004), it is currently unknown whether ORNs are differentially distributed in cartilaginous fishes, nor whether higher densities of ORNs would be expected in epithelial regions of the olfactory rosette that are exposed to higher hydrodynamic flow rates. A topographic organization (also termed odotopic or chemotopic organization), in which the axons of widely distributed ORNs across the rosette converge onto specific sets of glomeruli in the olfactory bulb, based on the class of chemicals they detect, has been widely accepted in teleosts (Riddle and Oakley, 1991; Baier et al., 1994; Hara and Zhang, 1996, 1998; Friedrich and Korsching, 1998; Morita and Finger, 1998; Laberge and Hara, 2001; Nikonov and Caprio, 2001; Hansen et al., 2003; Sato et al., 2005; Hamdani and Døving, 2007). However, in chondrichthyans, the organization of primary olfactory projections into the olfactory bulb is currently debated. Some studies have suggested a topographic organization (i.e., projections based on function as in teleosts) in the olfactory bulb of the small spotted catshark *Scyliorhinus canicula* (Ferrando et al., 2009), while others propose a somatotopic organization (i.e., projections based on epithelial location) in four selachians (sharks) and two batoids (rays) (Daniel, 1934; Dryer and Graziadei, 1993; Meredith et al., 2013).

Moreover, the chondrichthyan olfactory bulb has been shown to be compartmentalized, with anatomically distinct lateral and medial olfactory bulb regions (Dryer and Graziadei, 1993). Some species even have physically separated “hemi-bulbs,” such as in the lemon shark *Negaprion brevirostris* (Northcutt, 1978; Meredith et al., 2013), the tiger shark *Galeocerdo cuvier* (Yopak et al., 2015), the blue shark *Prionace glauca*, and the silky shark *Carcharhinus falciformis* (Lisney and Collin, 2006). Other species have dual swellings apparent from the olfactory bulb surface morphology, such as the epaulete shark *Hemiscyllium ocellatum*, the giant chimaera *Chimaera lignaria* (Yopak et al., 2015), the Greenland shark *Somniosus microcephalus*, and the Pacific sleeper shark *S. pacificus* (Yopak et al., 2019), while others have an aggregation of less apparent swellings on long, cylindrical olfactory bulbs, as in the bonnethead shark *Shyrna tiburo* (Northcutt, 1978), and other *Sphyrna spp.* including the hammerhead shark *S. lewini* (Yopak et al., 2015) and some batoids (Meredith et al., 2013). Such compartmentalization may have functional significance in relation to the segregation of odor processing in this region of the olfactory system but this remains to be tested (Dryer and Graziadei, 1993; Meredith et al., 2013; Yopak et al., 2015). Considering the existing evidence for some level of morphological (Dryer and Graziadei, 1996) and functional regionalization (Ferrando et al., 2009) in the chondrichthyan olfactory bulb, the current study seeks to determine how similar the ORN populations and primary olfactory projections in the olfactory pathway of a representative species of elasmobranch are to those of teleosts. Specifically, it addresses the potential for differential distribution of ORNs, as found in some teleosts and tetrapods.

Here, we anatomically identify ORN types and their distribution in the rosette of the brownbanded bamboo shark, *Chiloscyllium punctatum* (Müller and Henle, 1841). Specifically, we use G-protein immunohistochemistry, combined with light and electron microscopy to characterize the ORN morphotypes and assess the density and distribution of these types across four regions of the rosette (two medial and two lateral). We also ran three-dimensional simulations of the fluid dynamics through the olfactory cavity, using a surface model of the head, nares, and rosettes obtained through X-ray micro-computed tomography, to assess whether there is a correlation between the distribution of ORNs and the hydrodynamics of the inhalant water. Based on the lateral segregation of primary inputs projecting into the glomerular clusters within the olfactory bulb (Camilieri-Asch et al., 2020b), we hypothesize that there will be different densities of specific ORN types in the medial and lateral regions of the rosette, which could be correlated with the differential flow dynamics in the nasal cavity. We demonstrate that the use of these multimodal imaging techniques provides an integrated perspective of the functional organization of the olfactory pathway in cartilaginous fishes.

## Materials and Methods

### Specimens

Eight juvenile specimens of the brownbanded bamboo shark, *Chiloscyllium punctatum*, ranging from 23.0–36.5 cm in total length and 33.56–114.00 g in body weight, were used in this study. Other morphometric data (sex, total length at the subterminal notch, pre-caudal, and fork lengths) were also recorded (Table 1). All specimens were acquired as juveniles or egg cases from an approved commercial breeding colony in Queensland, Australia, bred or kept in aquaria at The University of Western Australia (UWA), and euthanized under Animal Ethics Approval No. RA/3/100/1153. All specimens were deeply anesthetized with tricaine methanesulfonate salt (MS-222; 250–500 mg/l in seawater) buffered to pH 7.2 with an equal concentration of sodium bicarbonate (NaHCO_3_), and transcardially perfused with a modified Karnovsky’s fixative solution (2.5% glutaraldehyde, 1% paraformaldehyde, 4% sucrose and 1% dimethyl sulfoxide in 0.13 M Sorensen’s phosphate buffer, pH 7.4) or 4% paraformaldehyde in 0.1 M phosphate buffer (Table 1). The head of each specimen was severed behind the second cervical vertebra and post-fixed by immersion in the same fixative solution as used in the perfusion, then stored at 4°C for 10 days prior to further processing. All procedures were carried out in strict accordance with the ethical guidelines of UWA and the Australian Code of Practice for the Care and Use of Animals for Scientific Purposes (8th Ed., 2013). Sample preparation and imaging were completed at the Centre for Microscopy, Characterisation and Analysis (CMCA) at UWA.

**TABLE 1:** Characteristics from all the specimens of *Chiloscyllium punctatum* (CP) used in this study.

Specimen	Sex	Maturity	BW (g)	TL (cm)	TLn (cm)	PCL (cm)	FL (cm)	Fixative	Use
CP1	F	im	45.24	25	24.5	18	19.5	Karnovsky*	CT + Sim
CP2	F	im	33.56	23	22.5	16.5	18.5	Karnovsky*	IHC
CP3	M	im	45.65	26	25.5	18.5	20.5	Karnovsky*	LEM + IHC
CP4	F	im	89.00	31	30.5	23	25	Karnovsky*	CT
CP5	M	im	114.00	36.5	35	25.5	28	Karnovsky*	CT
CP6	F	im	92.00	32	31.5	23	25.5	Karnovsky*	CT
CP7	M	im	46.60	24.5	24	19	19.5	4% PFA	LEM + IHC
CP8	M	im	34.30	23.5	23	18	20	4% PFA	LEM + IHC

F, female; M, male; im, immature; BW, body weight in gram; TL, total length in centimeters; TLn, total length at the subterminal notch; PCL, pre-caudal length; FL, fork length; *modified Karnovsky’s fixative i.e., without sodium cacodylate (2.5% glutaraldehyde, 1% paraformaldehyde, 4% sucrose and 1% dimethyl sulfoxide in 0.13 M Sorensen’s phosphate buffer); PFA, paraformaldehyde; CT, diceCT imaging; Sim, computer simulations based on rendered 3D model of the specimen’s head and olfactory cavities; IHC, immunohistochemistry; LEM, light and electron microscopy.

### Diffusible Iodine-Based Contrast-Enhanced Computed Tomography (diceCT)

To obtain a general overview of the organization of the olfactory system *in situ*, from peripheral organs (rosettes) to the central nervous system (brain), the heads of four fixed specimens of *C. punctatum*, namely CP1, 4, 5, and 6 (see Table 1), were imaged with an X-ray microscope (Versa 520 XRM, Zeiss). Following the protocol described in Camilieri-Asch et al. (2020a), specimens were placed in 300 ml of an aqueous solution of Lugol’s iodine (I_2_KI) – 1% w/v I_2_, 2% w/v KI in deionized water (dH_2_O) (Culling, 1963) – on a plate stirrer at room temperature (22°C constant), for 240 h (10 days). Specimen CP1 was stained for longer (336 h or 14 days) to provide extra contrast of specific head regions (skin, nares, and olfactory cavities), which facilitated segmentation and preparation of the data as surface mesh models for the study of fluid dynamics (see below). The stain was replaced with a fresh solution every 24 h. The olfactory organs (rosettes) and the rostral region of the forebrain (including olfactory bulbs, peduncles, and anterior telencephalon) were scanned using forebrain scanning parameters as outlined in Camilieri-Asch et al. (2020a) (CP1: voltage, 80 kV; amperage, 7 μA; filter, LE3; source, −45 mm; detector, 103 mm; isotropic voxel size, 11 mm; objective, 0.4X; binning, 1; exposure time, 5 s.; projection number, 2501; scanning time, 380 min.; CP4-6: voltage, 80 kV; amperage, 7 μA; filter, LE3; source, −54 mm; detector, 103 mm; isotropic voxel size, 11.88 mm; objective, 0.4X; binning, 1; exposure time, 5 s.; projection number, 2501; scanning time, 393 min.). All specimens were scanned in air, apart from CP1, which was scanned in water after being placed in a vacuum oven overnight to help separate individual lamella for later segmentation of the rosette.

For CP4-6, the contrast levels observed in the head and within the brain allowed us to differentiate nervous tissue from other tissues (epithelial, connective, and muscular). This approach enabled us to segment, i.e., label, the olfactory rosettes, primary projections and olfactory bulbs, following the methods described in Camilieri-Asch et al. (2020a, 2020b), using the software Avizo (v9.2.0, Thermo Fisher Scientific, United States). Specifically, this was achieved by cropping down to the region of interest, applying a non-local-means (NLM) filter to attenuate noise, and then interactive labeling using the suite of tools in the segmentation editor. This yielded two label images: one corresponding to the rosettes, olfactory bulbs, peduncles, and anterior telencephalon, and the other to the glomeruli. Each label image was subsequently used to mask the corresponding regions of interest from the NLM-filtered image, to facilitate visualization (TIFF images and MPEG movies).

### Light and Electron Microscopy

Brains and olfactory organs (rosettes) from specimens CP3, 7, and 8 were surgically exposed (see Table 1). For each specimen, assuming bilateral symmetry, the left olfactory rosette was removed and retained for scanning electron microscopy and immunohistochemistry, while the right olfactory pathway (rosette, olfactory bulb, peduncle) was used for light and electron microscopy.

To characterize the olfactory receptor neurons (ORNs) in the olfactory epithelia of *C. punctatum*, we used light microscopy (LM) to observe transverse sections of olfactory lamellae at low magnification (40–400×). Following sample preparation detailed in Camilieri-Asch et al. (2020c), the anterior right olfactory pathway (rosette and anterior portion of the olfactory bulb) was isolated and dissected into smaller samples ca. 1–2 mm in thickness. Tissue samples were post-fixed with 1% osmium tetroxide (OsO_4_) in Sorensen’s phosphate buffer for 2 h, dehydrated and resin-infiltrated using a Lynx^TM^el (Electron Microscopy Sciences, United States) tissue processor and embedded in resin (25 g Procure, 15 g Araldite and 55 g DMSA). Resin blocks were cured at 60°C in an oven for 24 h. Semi-thin sections (500 nm) of the olfactory lamellae were cut with glass knives using an ultra-microtome (Leica, EM UC6). Sections were floated onto glass slides, stained with 1% Toluidine Blue in 5% boric acid, and mounted with Permount^TM^ mounting medium (ProSciTech, IA019). Images (2464 × 2056, RGB Color, TIFF format, uncompressed) were acquired using a light microscope (Zeiss Axioskop 2 plus) mounted with an Axiocam 305 color (Carl Zeiss Microscopy, Germany) digital camera.

To assist in the differentiation of ORN types, transmission electron microscopy (TEM) was used to image transverse sections of the epithelium at higher magnification (2,000–20,000×). Ultrathin sections (100 nm) of the olfactory lamellae were cut using a diamond knife (Diatome, knife No. MX5582, ultra, 3.5 mm, 35°, for 6°CI angle) and mounted onto copper grids (200 mesh thin square bars, ProSciTech). The gridded sections were stained with lead citrate, rinsed with dH_2_O and blotted dry with filter paper. Images were acquired (4008 × 2672, 16-bit, dm3 format, uncompressed) using a JEOL2100 TEM fitted with an Orius SC1000 digital camera (120 kV acceleration voltage).

To gain an overview of the apical surfaces of the olfactory mucosa and complement previous observations using LM and TEM, we used scanning electron microscopy (SEM) both at low (20–200×) and high (1,000–62,000×) magnifications. Stacks of 2–3 lamellae (lamella pair) were dissected out of left olfactory rosettes from specimens CP7 and CP8, then washed in phosphate buffered saline (PBS) and dehydrated in a graded ethanol series using a protocol in the microwave (PELCO Biowave microwave fitted with PELCO coldspot) using two step repeats, with 40 s per step at 250W and no vacuum (PBS wash, dH_2_O wash, 30%, 50%, 70%, 95%, 100% ethanol). Samples were then critical-point dried in liquid CO_2_ (Polaron E3000 critical point drier, at 1,100psi and 31.1°C) for 1 h. Samples were mounted on a 12.6 mm pin stub (ProSciTech, G040) lined with a 12-mm carbon tab (ProSciTech, IA023), and coated with a conductive layer of platinum (5 nm) and carbon (10 nm) for optimized imaging at higher magnification (up to 70,000×) and low voltage (5 kV). SEM imaging was performed using a 1555 VP-FESEM (Zeiss, Germany). Images (1024 × 768, RGB Color, uncompressed) were exported as TIFF files.

### Immunohistochemistry and Confocal Microscopy

**FIGURE 1:**
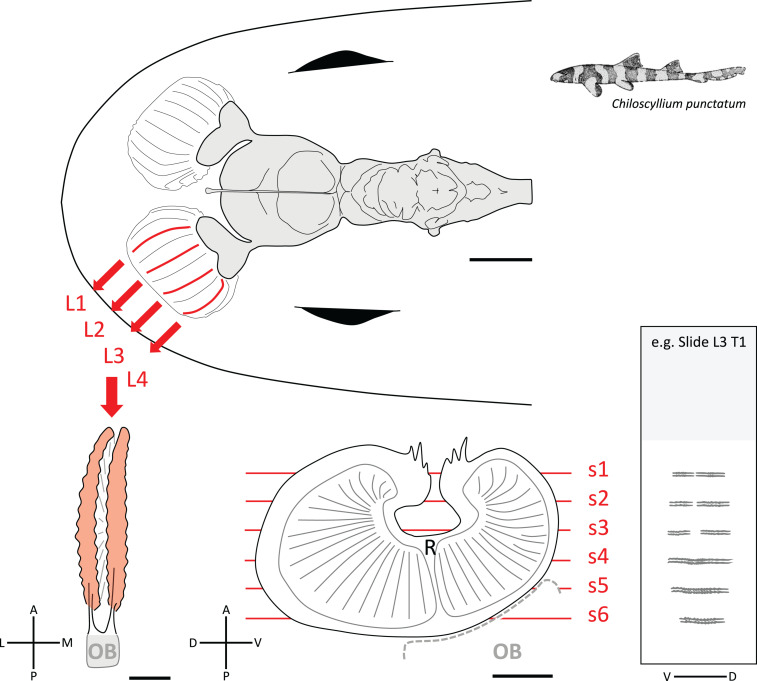
Diagram of the brain and olfactory pathway in *Chiloscyllium punctatum* illustrating the sampling used for immunohistochemistry (IHC). **(Top)** Dorsal view of the brain (gray) and peripheral olfactory organs, including olfactory rosettes and olfactory nerves (white) in *C. punctatum*, showing the four locations (two medial, two lateral) in the left olfactory rosette (red lines) from where lamella pairs (L1-4) were collected for IHC. **(Bottom left)** dorsal view of one lamella pair; once the lamella pair was dissected from the olfactory rosette, the remaining olfactory bulb (OB) was then removed. **(Bottom middle)** lateral view of one olfactory lamella (the lamellae stack has not been drawn in perspective to simplify the view), made up of two single folds bearing many secondary folds (solid gray lines) and separated by a central raphe (R). The red lines show the approximate positions of the cryosections cut for IHC labeling and confocal microscopy imaging. **(Bottom right)** representative slide illustrating the six cryosections collected for each lamella pair, under each of the five treatments (T, or marker used), and arranged on a microscope slide. L, lateral; M, medial; A, anterior; P, posterior; D, dorsal; V, ventral. Scale bars = 5 mm (top), 1 mm (bottom).

To assess the presence of different ORN types in *C. punctatum*, antisera directed against five different G-protein α-subunits were used, referred to as five treatment for analysis purposes (Table 2). Tests were conducted to ascertain whether ORN subtypes were distributed differently across and within olfactory lamellae, specifically between medial and lateral parts of the rosette. Four pairs of lamellae (two medial, two lateral) were dissected from each of the left olfactory rosettes of specimens CP2, 3, 7, and 8 (Figure 1). Lamella pairs were placed in 15% glucose in Sorensen’s buffer overnight, embedded and flash frozen in optimal cutting temperature (OCT) compound, and serial cryosectioned transversally using a Leica CM1900 cryostat. Every 50th section (i.e., every 600 microns) was collected onto a SuperFrost^TM^ Plus slide for each treatment (i.e., antiserum used). Six 12 μm cryosections per lamella pair were collected on each treatment slide (five slides of six sections per lamella pair) (Figure 1). Slides were placed in 0.1% Sudan Black B (C_29_H_24_N_6_) diazo dye in 70% ethanol for 5 min, to quench tissue autofluorescence, and rinsed in Tris-buffered saline (TBS) (4.3 g Trizma Base, 3.1 g NaCl in 500 ml dH_2_O, pH 7.5). All slides were left in blocking solution [10% fetal calf serum (FCS), 10% normal goat serum (NGS), 0.1% bovine serum albumin (BSA), 0.1% Triton X-100, in TBS] for 30 min. After three washes in TBS, slides were incubated for 30 min with mouse monoclonal antibody diluted in blocking solution (Gα_s/olf_ 1:100, Gα_*o*_ 1:100, Gα_q/__11__/__14_ 1:100, Gα_i/__1__/__2__/__3_ 1:100, Gα_i–__3_ 1:100; stock concentrations 200 μg/ml, Santa Cruz Biotechnology, Inc., Santa Cruz, CA, United States; Table 2). Slides were washed three times with TBS, followed by incubation with anti-mouse fluorescently labeled secondary antibody (Alexa Fluor^®^ 568 1:500; Thermo Fisher Scientific Inc., United States) and DNA marker (Hoechst 33342, 2.5 ng/ml) for 30 min. Slides were then washed with TBS and mounted. Negative control slides were placed in the blocking solution for 30 min, rinsed with TBS and mounted. Secondary negative controls were placed in the blocking solution for 30 min, rinsed with TBS, left in the secondary antibody and fluorescent dye solution for 30 min, rinsed and mounted. To help identify and characterize cell types, two neuronal markers, Protein Gene Product 9.5 (PGP 9.5; stock concentration 0.113 mg/ml, Abcam, United Kingdom) and purified Tubulin β3 (TUBB3; stock concentration 1 mg/ml, BioLegend, CA, United States), were also tested using the same protocol and working dilutions (Table 2).

**TABLE 2:** Primary antisera used.

Antisera	Supplier	Cat. No.	Lot No.	Source/raised against
Gα_s/olf_ (A-5)	Santa Cruz	sc-55545	G1116	aa. 82-381 mapping at C-terminus of Gα_s/olf_ of human origin
Gα_*q/11/14*_ (G-7)	Santa Cruz	sc-365906	K1017	aa. 60-359 mapping at C-terminus of Gα_11_ of human origin
Gα_ i–1/2/3_ (37)	Santa Cruz	sc-136478	H2317	aa. 90-108 of Gα_ i–1_ of human origin
Gα_i–3_ (H-7)	Santa Cruz	sc-365422	F0217	aa. 339-354 at the C-terminus of Gα_i–3_ of rat origin
Gα_*o*_ (A2)	Santa Cruz	sc-13532	B1213	Gα_*o*_ of bovine origin
PGP9.5	Abcam	ab108986	–	Recombinant rabbit anti-PGP9.5 antibody [EPR4118]
TUBB3	BioLegend	801201	B2092	Raised against microtubules derived from rat brain

*Mouse monoclonal antibodies. Aa, amino acid.*

Prior to the study, trials were carried out to test the presence and level of labeling of the olfactory epithelium. Primary and secondary controls confirmed that the labeling observed was positive across all Gα antisera tested. However, no positive labeling was observed for PGP 9.5. Furthermore, TUBB3 labeled cilia inconsistently (including cilia from supportive cells, although this antiserum is a neuronal marker targeting neuronal tubulin in mammals), thus providing from conducting double-labeling experiments with Gα antisera to further characterize the different ORN morphotype in our species. It is worth noting that the tissue autofluorescence was high across specimens, even though we attempted to quench it with 0.1% Sudan Black B.

Sections of whole lamellae were imaged at 20 × (NA 0.75, Nikon, Japan) on a Nikon A1Si confocal microscope (inverted motorized microscope with a 32-channel spectral detector) running NIS-Elements AR software (v4.60.00). Images were captured using either the galvanometer or resonant scanners, with 405 and 561 nm lasers using PMT or GaAsP detectors respectively. Images were acquired as z-stacks (step size of 2 μm) to ensure the full thickness of the section was captured. Identified labeled cells were also imaged at high magnification (*n* = 10–20 cells per treatment) (100 × oil immersion, NA 1.49) on the Nikon A1Si confocal microscope. All images captured used identical microscope settings per treatment group to prevent imaging bias. For analysis, all images used in this study were saved as nd2 files (three channels, 16-bit each channel).

Each low magnification 20 × image stack was processed using one of two purpose-written FIJI scripts (Image J macro language – see Supplementary Materials 1 and 2) to assess cell density from 2D maximum intensity projections in each channel. The first script is designed for stacks where the labeling is non-prevalent (Supplementary Material 1). It facilitates manual counting of labeled cells in a selected area of tissue. The second script is designed for stacks where the labeling is prevalent and relatively homogeneous (Supplementary Material 2). It facilitates the selection of five regions of interest (ROIs), interactive estimation of the number of labeled cell nuclei in each ROI based on the detection of intensity maxima, and the computation of the mean label intensity in each ROI. Of these two measurements, the latter was used in the data analysis. It is the mean amount of labeling per unit pixel area (pixel size = 1.2581 μm) and is a proxy for the density of labeled cells. It was chosen in preference because of the subjectivity and uncertainty associated with the maxima detection approach. The density of cells (non-prevalent labeling) and/or the mean intensity per unit pixel area (prevalent labeling) were then analyzed. High magnification images were also captured to characterize the labeled cell types across treatments by assessing the cell shape, size and position in the epithelia, as well as nucleus size and position in the cell body. These images were exported as TIFF files (16-bit, uncompressed) for each color channel (DAPI and Alexa 568), and then merged, using NIS-Elements AR.

### Computational Fluid Dynamics

The rendered volume of the μCT-scanned specimen (CP1) was processed using the software Avizo (v9.2.0) to generate a model surface of the shark head and olfactory cavities. This model was used to simulate internal hydrodynamics of the inhalant water flow through the olfactory cavities.

#### Surface Mesh Generation

The raw volume data (TXM file) was imported into Avizo and filtered (non-local means filter) to reduce image noise and assist with segmentation. The head (skin) and internal olfactory cavities (including the lining lamellae forming the rosettes) were segmented using the segmentation editor with a combination of manual thresholding methods and a succession of erosion/dilation operations to define internal (biological tissue) and external regions. It is important to note that we were not able to manually separate all individual lamella, most of which were clumped together at their anterior edges, especially in the middle region of the rosette, even after using the vacuum oven (i.e., to degas water). As such, future studies should consider staining the negative space (i.e., the surrounding medium) instead of the specimen, and increase the viscosity of the medium. A single binary connected component representing the external surface of the sample including mouth and nasal/olfactory rosettes, was then created. A triangular mesh of the tissue surface was then generated (smoothing parameter set to 5) and exported as a STL file, which is a suitable format for further mesh refinement and hydrodynamic modeling.

#### Mesh Modification

The nostril flaps and barbels were digitally repositioned to correct for sample deformation that occurred during fixation and storage. This repositioning was guided by photographic information taken from live animals by VCA and the observed *in vivo* symmetry of the external morphology was used to reflect their natural appearance. The nostril flaps on both sides of CP1 were widened and barbels were straightened. Each of these features were manually morphed using the “soft transform” linear deformation tool in Meshmixer (v3.4.35, Autodesk, San Rafael). The sample can be seen before and after manual morphing in Supplementary Figure 3.

#### Mesh Generation and Refinement

**FIGURE 2:**
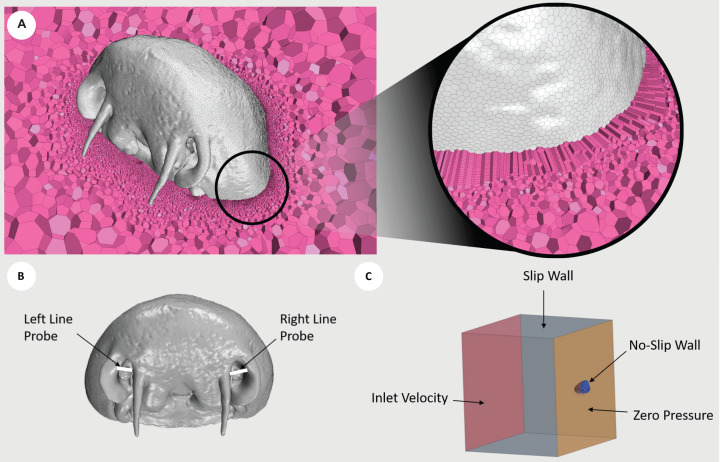
Diagrams illustrating the conditions used. **(A)** Cross section of CP1 displaying the internal polyhedral meshing elements (pink), with a zoomed view demonstrating the implementation of prism layer elements. The mesh displayed was generated using the fine mesh parameters. **(B)** Line probes (white) placed at the entrance to the nostril cavities of CP1. The average Reynolds number was extracted along each line probe. **(C)** Boundary conditions of inlet velocity (red), slip wall (gray), no-slip wall (blue), and zero pressure (orange), prescribed about the fluid volume surrounding CP1. Red, right lateral; green, right medial; yellow, left medial; blue, left lateral.

The morphed geometry surface mesh was imported into the commercial computational fluid dynamics (CFD) package STAR-CCM + (v12.06, Siemens, Berlin). To generate a mesh with adequate density and to ensure sufficient capture of the wall shear stress (WSS) and derived metrics, a mesh independence study was performed using the CP1 geometry experiencing highest inlet velocity conditions (120 cm/s). The grid convergence index (GCI), which is a measure of asymptotic convergence to a theoretical value for a parameter (Roache, 1994), was employed with the parameter of interest being surface averaged WSS (SAWSS). Results of the GCI of the SAWSS across each region (LR1 = 2.53%, LR2 = 1.78%, RR1 = 1.41% and RR2 = 0.23%) were all found to fall below 3%, which corresponds to a range that is considered to be sufficiently converged based on previous studies (Doyle et al., 2014). A summary of meshing parameters used in the mesh independence study are presented in Supplementary Table 4, with GCI calculated using the non-uniform refinement ratio formulation. The results for SAWSS across half regions of the rosettes along with the corresponding GCI value is presented in Supplementary Table 7. Based on this study, the final meshing parameters employed polyhedral mesh elements in conjunction with a layer of prism layer elements across the shark wall surfaces. As per the meshing conditions specified for the fine mesh in Supplementary Table 5, a target surface size of 0.15 mm was specified, along with 24 prism layers that were prescribed over 0.0015 m distance, generating a final volume mesh of 1.4 million control volume elements. A view of the mesh density can be seen in Figure 2A.

#### Fluid Assumptions

The continuum fluid was modeled as seawater, which was assumed to be incompressible with a constant density of 1024.81 kg.m^–3^, and as a Newtonian fluid with a constant viscosity of 0.001077 Pa.s^–1^. The flow was assumed to be steady and laminar. This assumption was verified by the average Reynolds number (Re) for the highest inlet flow rate (120 cm.s^–1^), which was measured at the inlet to the shark’s left (Re = 591) and right (Re = 321) nostrils, and exhibited values below the value for transition to turbulence (Rygg et al., 2013). The Reynolds number was calculated across line probes (Figure 2B).

#### Boundary Conditions

An inlet velocity condition was prescribed at the nostril inlet. Three simulations were constructed, with the boundary specified a value of velocity normal to its surface of 20, 60, and 120 cm.s^–1^. These velocity values were chosen based on previously reported cruising and critical swimming speeds for a range of demersal, bentho-pelagic shark species (Carrier et al., 2012; Ryan et al., 2015; Whitney et al., 2016). A zero-pressure outlet condition was employed at the chamber outlet and at the mouth boundary. Although this is an unrealistic specification at the shark’s mouth outlet (because the internal resistance of the shark’s passageways and gills would provide a natural backpressure), the *in vivo* value is unknown and hence the assumption of a zero-pressure outlet was prescribed. However, as this variable was held constant across inlet boundary conditions, the trends observed in hydrodynamic parameters between cases are still considered to be informative. The surfaces of the rostrum and rosettes were assumed to be rigid wall boundaries with specification of the no-slip WSS condition, with roughness assumed to be negligible. Fluid slip boundary conditions were assumed at the walls of the bounding chamber (Figure 2C).

#### Numerical Methods

Final simulations were solved using the steady segregated flow solver and employing a 1st order convection scheme. For both sides (left and right), data were collected at two levels; the entire olfactory cavity, and each cavity partitioned into lateral and medial regions, using a different set of probes for each level. All data were extracted after at least 2500 iterations, with normalized residuals for momentum and continuity observed to fall below 10^–4^ across all velocity cases. Simulations were performed on a Dell XPS laptop using 3 Intel i7-7700HQ 2.80 GHz cores.

### Statistical Analyses

Linear regression models were used to test for differences in the spatial distribution and density of labeled ORNs. Differences were compared between and within olfactory lamella pairs (*n* = 8, four per specimen) sampled in the rosette, between treatments (*n* = 5 markers), and between specimens fixed in PFA (*n* = 2 individuals), from which reliable labeling was obtained for all treatments. Any significant interaction terms identified were tested using Tukey’s *post hoc* tests to identify significant contrasts between variables. A two-way Analysis of Variance (ANOVA) was used to detect potential differences in hydrodynamic variables (mean velocity, pressure, wall shear stress and vorticity) between the medial and lateral sides of the olfactory cavities in one specimen in order to compare between the two regions over simulation times. All statistical analyses were conducted using “emmeans” package (Lenth et al., 2019) in R Core Team (2019).

## Results

### Olfactory Organs

**FIGURE 3:**
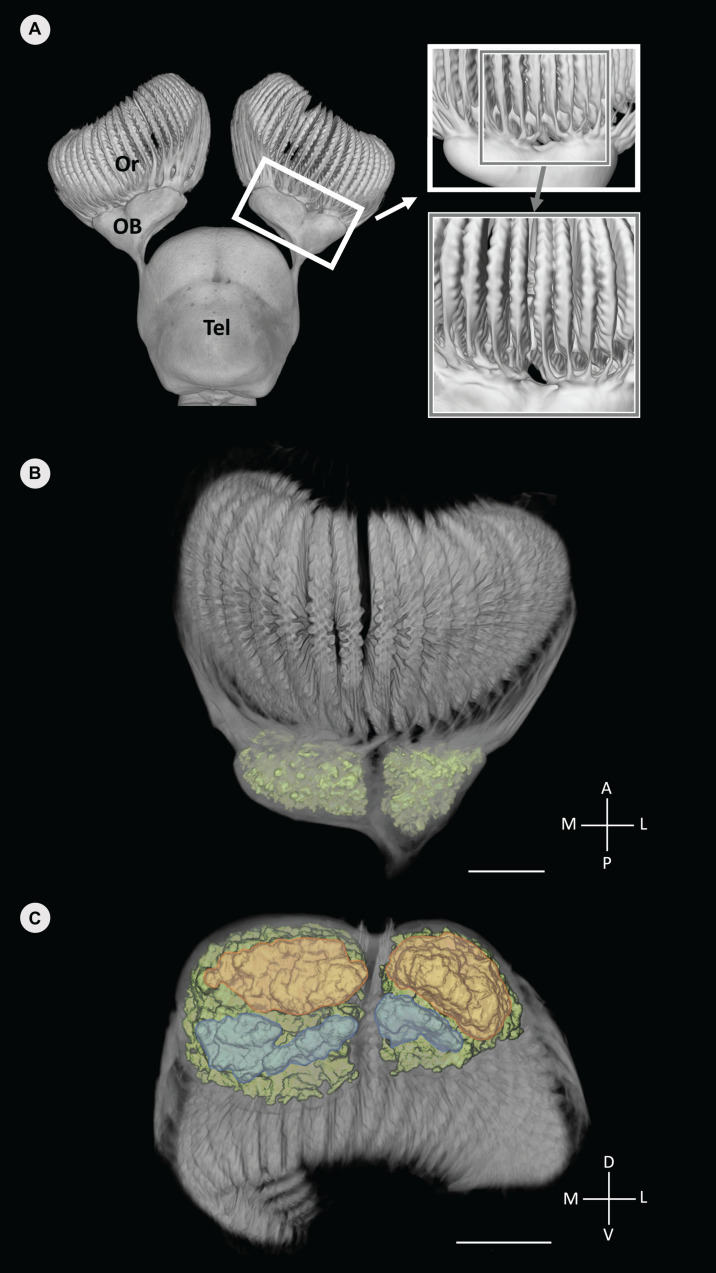
Volume rendered data of the anterior forebrain (olfactory rosettes, olfactory bulbs and telencephalon) of *Chiloscyllium punctatum* segmented in Avizo **(A)**, with the glomeruli clusters segmented and labeled in color (green) **(B,C)**. **(A)** Dorsal view of the olfactory pathway, highlighting the segregation of primary olfactory projections between lamellae from the medial and lateral sides of the rosette on magnifying windows (each window showing a view, rotated 15° left). **(B)** Dorsal view of the olfactory bulb (using a different color map to **B**), showing the distinct lateral and medial clusters of glomeruli (green). **(B)** Antero-posterior view (posterior in foreground) of the olfactory bulb and glomeruli clusters (color), showing the posterior part of each glomeruli cluster, which appear further divided dorso-ventrally, into dorsal (orange) and ventral (blue) areas (see Video 1 in Supplementary Material). Or, olfactory rosette; OB, olfactory bulb; Tel, telencephalon; M, medial; L, lateral; A, anterior; P, posterior; D, dorsal; V, ventral. Scale bars = 5 mm. Adapted from Camilieri-Asch (2019).

**FIGURE 4:**
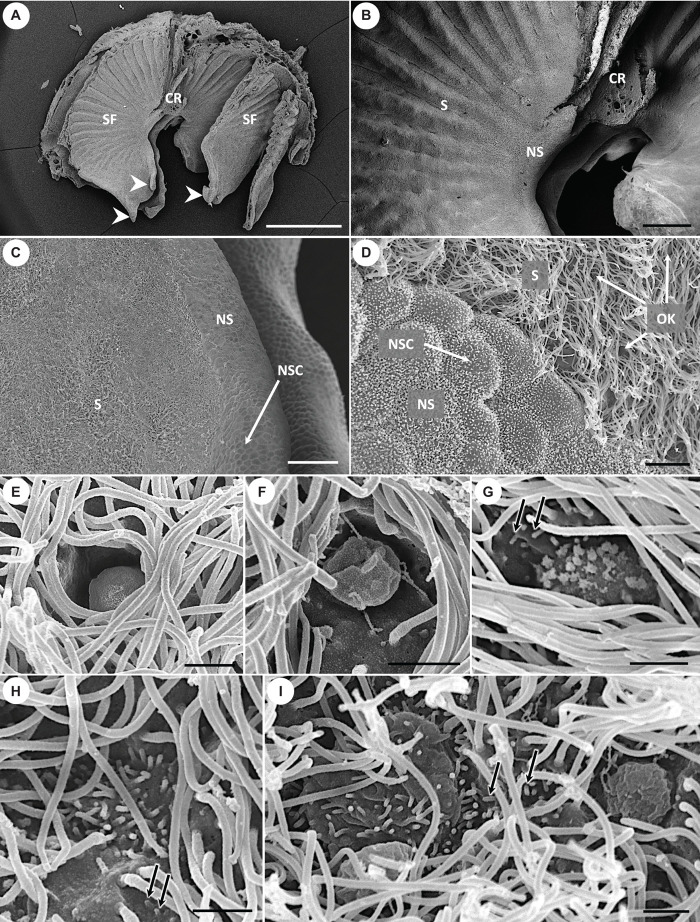
Scanning electron micrographs showing the morphology of olfactory lamellae in *Chiloscyllium punctatum* **(A–D)** and the diversity of apical knobs of olfactory receptor neurons (ORNs) in the neuroepithelium **(E–I)**. **(E)** Flattened apical knob, with a rugose surface and no visible protrusions. **(F)** Bulbous apical knob, with an amorphous, rugose surface and some filamentous protrusions. **(G)** Rounded apical knob with short, stubby protrusions. **(H)** Microvillous apical knob surface, with no tabular cap. **(I)** Large microvillous apical knob, protruding from the luminal surface in a rounded cap-like shape (left), and another example of bulbous apical knob with an amorphous, rugose surface and thin, long, “filament-like” protrusions (right). CR, central raphe; SF, single lamellar fold on either side of the central raphe; S, sensory epithelium; NS, non-sensory epithelium; NSC, non-sensory cell; OK, olfactory receptor neuron apical knob; white arrowheads, free-floating protrusions at the anterior part of the single folds; black arrows, microvilli also born by supporting cells in the sensory epithelium. Scale bars = 1 mm **(A)**, 200 μm **(B)**, 50 μm **(C)**, 5 μm **(D)**, 1 μm **(E–I)**.

**FIGURE 5:**
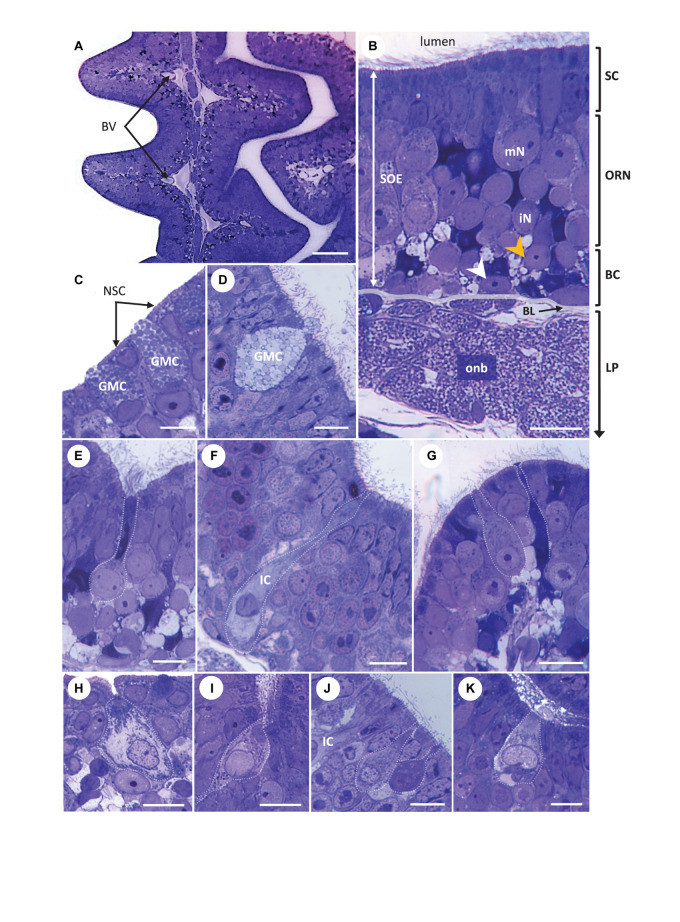
Light micrographs. Transversal semi-thin sections of the olfactory organ in *Chiloscyllium punctatum* (stained with Toluidine blue), showing the complementary arrangement between adjacent olfactory lamellae **(A)**, the pseudo-stratified organization of the olfactory mucosa **(B)** and different cell types found in the two olfactory epithelia **(C–K)**, including several olfactory receptor neuron (ORN) morphotypes in the olfactory neuroepithelium **(E–K)**. **(C,D)** Goblet mucous cells present in both the non-sensory epithelium at the periphery of olfactory lamellae **(C)** and within the sensory olfactory epithelium (SOE or neuroepithelium), filled with circular configurations **(D)**. A droplet of mucus forming in panel **(D)**. **(E)** Elongated ORN, with a round soma sitting in intermediate to low layer of the SOE, a large, round basal nucleus occupying most of the soma, and thin, extended dendrite ending in apical knob with undefined protrusions (unclear; it could bear microvilli and/or cilia), here located at the bottom of a trough between secondary folds. **(F)** Apparent ionocyte (IC), with a soma spanning the entire neuroepithelium, an intermediate to basal, fragmented nucleus, and a clear cytoplasm rich in organelles within the baso-lateral and apical regions. **(G)** Typical microvillous ORN, with a soma sitting in the intermediate layer of the neuroepithelium, a broad dendrite, and an apical knob bearing microvilli (left), here close to the top of a secondary fold. Another possible ORN morphotype, with a thinner dendrite and a tabular apical knob (right). **(H)** Crypt-like cell at the periphery between the two epithelium types, on the non-sensory epithelium side. **(I)** Crypt-like cell in the neuroepithelium, at the bottom of a secondary fold trough. **(J)** Teardrop (top) and pear-shaped (bottom) ORN morphotypes, both with narrow apical knobs. The somas of teardrop shaped cells usually sit lower than those of pear-shaped cells, although both types are found in the upper part of the neuroepithelium. A swelling is often present on the apical end of the dendrite in pear-shaped cells, as shown. Part of a ionocyte (IC) on the left-hand side. **(K)** Kappe-like neuron within a secondary fold trough, with a cup-shaped soma in the upper layer of the neuroepithelium, a large, basal nucleus and a short, thick dendrite, which apical knob bears short protrusions. BV, blood vessel; SOE, sensory olfactory epithelium (or neuroepithelium); SC, supporting cell layer; ORN, olfactory receptor neuron layer; BC, basal cell layer; LP, *lamina propria*; mN, mature neuron; iN, immature neuron; BL, basal lamina; onb, olfactory nerve bundles wrapped by ensheathing cells; white arrowhead, horizontal basal cell; orange arrowhead, global basal cell; NSC, non-sensory cell; GMC, Goblet mucous cell; IC, ionocyte. Scale bars = 50 μm **(A)**, 20 μm **(B,E)**, and 10 μm **(C,D,F–J)**.

The paired olfactory openings of the brownbanded bamboo shark, *Chiloscyllium punctatum*, bear a barbel on their medial side (Figures 2A,B) and are positioned directly frontal to the mouth on the ventral side of the rostrum (Figure 2B). In each olfactory cavity, the olfactory organ (rosette) comprises approximately 40 single lamellar folds (for specimens from this size range – Figure 3A), which arise from either side of a central raphe, and are not necessarily aligned (Figure 4A). Lamellae are supported by a cartilaginous capsule behind the rosette wall, apart from free-floating protrusions (Figure 4A) on the anterior part of each single fold, which can interlock. Lamellae are largest in the central region of the rosette, tapering and becoming smaller toward the outer regions. Secondary folds are triangular (Figures 4A,B) and interdigitate with adjacent lamellae (Figures 3A, 5A). The thickness of the olfactory mucosa increases from anterior to posterior parts of the lamellar folds (Figure 4A). In this species, the olfactory lamellae are mostly covered by sensory olfactory epithelium (SOE or neuroepithelium), i.e. SOE is present both on the secondary folds and in the troughs (Figure 4B). The non-sensory epithelium is concentrated at the edges of the lamellar folds (Figures 4B,C). Non-sensory cells are large, rounded cells, bearing only short microvilli (Figures 4C,D), as seen in other species. The SOE was largely populated with supporting cells, which bear both long cilia and some microvilli on their apical surface, and surround olfactory receptor neuron (ORN) apical knobs (Figures 4D–I). Several apical knobs of ORNs were observed, dispersed amongst supporting cells, but were often obscured by the high density of cilia from the surrounding supporting cells (Figures 4D–F).

### Lateral Segregation of Primary Projections Into a Compartmentalized Olfactory Bulb

Volume rendered data from μCT scans of the head of *C. punctatum*, which include the peripheral olfactory organs (rosettes), their primary projections, and the frontal part of the forebrain (olfactory bulbs, peduncles, and anterior telencephalon), reveal an anatomical partitioning between medial and lateral sides, at the first level of convergence in the olfactory pathway. Externally, primary projections (i.e., axonal bundles) originating from medial and lateral olfactory lamellae appear to project separately into the medial and lateral swellings of the olfactory bulb (Figure 3A). Internally, two distinct clusters of glomeruli, corresponding to the two segregated regions, are observed in each olfactory bulb, as shown in Figure 3B. From a frontal orientation, the posterior portion of both clusters is sub-divided into dorsal and ventral regions (Figure 3C and Supplementary Video 1). The voxel intensities between and within individual glomeruli prevented further segmentation of individual glomerular structures within each cluster.

### Histology of the Olfactory Epithelia

**FIGURE 6:**
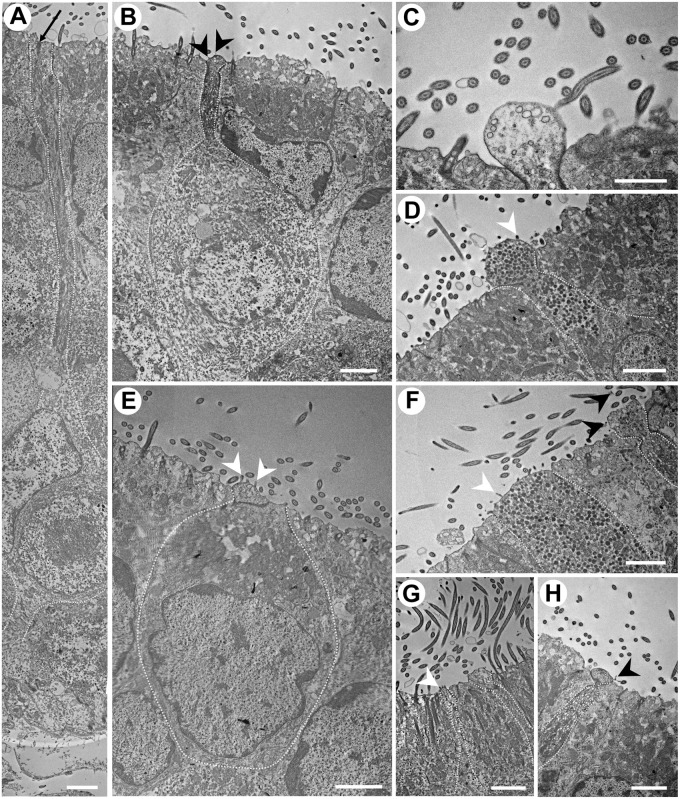
Transmission electron micrographs of the neuroepithelium of *Chiloscyllium punctatum*, showing several olfactory receptor neuron (ORN) morphotypes. **(A)** Elongated ORN, with a long and thin dendrite. The black arrow indicates part of a cilium rootlet. **(B)** Pear-shaped ORN, with a bulbous, protruded apical knob that bears thin filamentous protrusions, and a thin dendrite, constricted underneath the apical knob. The dendrite contains both electron-dense and electron-lucent vesicles. The dendritic swelling is not visible on the same plane as the apical knob showing the few, thin filamentous protrusion it bears on this section – cf. Figures 3F,I (right). **(C)** Smaller, bulbous apical knob, with a smooth surface and electron-lucent vesicles. **(D)** Suspected teardrop-shaped ORN, with a rounded, protruded knob bearing microvilli and a short, short dendrite thickening toward the apical soma. Numerous electron-dense vesicles are present in its dendrite. **(E)** Kappe-like ORN, with a cup-shaped soma very close to the apical surface of the neuroepithelium, a very large nucleus occupying most its base, an organelle-dense apical soma, a very short, thick dendrite, and a larger apical knob, protruding slightly and bearing microvilli. **(F)** Broad, tabular apical knob of a microvillous ORN (left). Numerous electron-dense vesicles are present in its dendrite. A possible pear-shaped ORN (right), with a narrow, constricted dendrite below an amorphous, protruded apical knob with thin, filament-like protrusions (black arrows), and both electron-dense and electron-lucent vesicles in the dendrite. **(G)** Type of ORN bearing microvilli on its apical knob (left) and a possible pear-shaped ORN (right). **(H)** Dendrite and apical knob of a pear-shaped ORN, with a protruded, round apical knob bearing thin, “filament-like” protrusions (cf. Figure 3I), a constricted apical dendrite, a dendritic swelling slightly visible on this section plane, and electron-dense and -lucent vesicles in its dendrite (cf.). White arrowheads, microvillus; black arrowheads, filamentous protrusions. All scale bars = 2 μm, except **(C)** 1 μm.

The SOE or neuroepithelium of *C. punctatum* is pseudo-stratified, with three main cell layers from lumen to basal lamina (Figure 5B). The most superficial cell layer contains mainly ciliated cuboid, columnar cells (supporting cells), as well as apical dendrites of bipolar sensory neurons (i.e., ORNs), some ORN somas (which sit within the upper SOE), and goblet mucous cells (Figure 5D). The following layer comprises the somas of other ORNs, while the basal cell layer below contains horizontal and global basal cells, as well as progenitor cells. Some ionocytes, which are large, rather columnar cells with a clear cytoplasm, spanning the entire SOE thickness, are also observed (Figure 5F). Formerly called chloride cells, ionocytes are found within the olfactory mucosa, gills and/or skin of various fishes, and have a role in maintaining an optimal osmotic, ionic, and acid-base balance. Ionocytes have been documented in the olfactory mucosa of three other elasmobranch species to date (Ferrando et al., 2006a, 2016, 2017; Ferrando, 2008). The non-sensory epithelium includes mainly non-sensory cells (i.e., non-ciliated columnar cells bearing small microvilli), mucous cells, and progenitors of both cell types (Figure 5C). The underlying *lamina propria* contains olfactory nerve bundles surrounded by connective tissue and blood vessels (Figures 5A,B). At least five (and possibly up to six) morphologically distinct ORN types are observed in the SOE, as visualized using SEM, LM, and TEM (Figures 4–6, respectively).

Long ORNs are characterized by an elongated shape, which spans the entire olfactory neuroepithelium, with a soma situated in the lower layers of the SOE, a circular nucleus occupying most of the soma, a long, thin dendrite and short or partial rootlets present beneath the apical membrane (Figure 6A), which could indicate the presence of cilia projecting from a narrow olfactory knob. This morphotype shows some similarities with ciliated ORN morphotypes present in other vertebrates. However, as no ciliated ORNs have been identified in any chondrichthyan fish to date, the presence of a form of ciliated morphotype in this species can only be suggested (Figures 5E,F, 6A).

Microvillous ORNs are vertically elongated, with an enlarged oval soma generally sitting in the medial layer of the SOE, and a smaller, circular nucleus at the base of the soma (Figure 5G). These cells have a thicker, columnar dendrite, with a slightly protruded apical knob bearing microvilli (Figures 4H, 5G, 6F).

Rare crypt-like ORNs are found in the upper layer of the SOE and are characterized by a large, clear, ovoid soma, a large foliose nucleus, and protrusions (here likely sectioned, i.e., on different planes) at their apical surface, within a crypt-like depression (Figures 5H,I). Very few cells of this type were found within the SOE, and only on one occasion at the periphery between the two epithelia types on the non-sensory side (Figure 5H).

Up to three other microvilli-bearing cells were observed. Kappe-like neurons are identified in the upper layer of the SOE and possess microvilli on their apical surface (Figures 4H, 6E). They have a large, cup-shaped soma, which contains a large, horizontally oval nucleus at its base, and a short, thick dendrite (Figures 5K, 6E). Pear-shaped and teardrop-shaped morphotypes are also located in the upper layer of the SOE, but the soma of teardrop-shaped cells lie slightly lower than pear-shaped cells (Figure 5J). Teardrop-shaped ORNs have a relatively small and circular nucleus located at the base of the cell soma, and a narrowing dendrite projecting to a rounded, protruded apical knob bearing microvilli (Figures 4G, 6D). Pear-shaped cells have a short, thin dendrite, which displays a swelling (observed to be at various distances along its length), and a constriction just below the apical knob, which thus appear rather tabular on sections (Figures 5G,J, 6F,H). The knob is generally ovoid but has an amorphous rugose surface, which bears filamentous protrusions (Figures 4F,I (right), 6B,F,H). These “filaments” are not numerous but are found extending away from the vicinity of the knob between the long cilia from surrounding supporting cells (Figure 4I), and sometimes appeared to be branching (Figure 6H). Their size (thickness, length) and structure could resemble those of filamentous actin (or actin filaments); although, the nature of these extremely thin protrusions was not further characterized in this study and will need to be confirmed.

### G-Protein Immunohistochemistry

We identified populations of different cells showing a positive immunoreactivity (–ir) for each antiserum. Four antisera showed non-prevalent labeling (Gα_s/olf_, Gα_q/__11__/__14_, Gα_i–__1__/__2__/__3_, Gα_i–__3_) and one was highly prevalent (Gα_*o*_). Although not histologically or immunohistochemically characterized in this study, the labeling of small, oval cells in the bottom layer of the neuroepithelium (just above the *lamina propria*), observed for each antiserum used, is referred to as “possible progenitor cells,” based on their position, size and shape on light microscopy observations (cf. Figure 5B).

**FIGURE 7:**
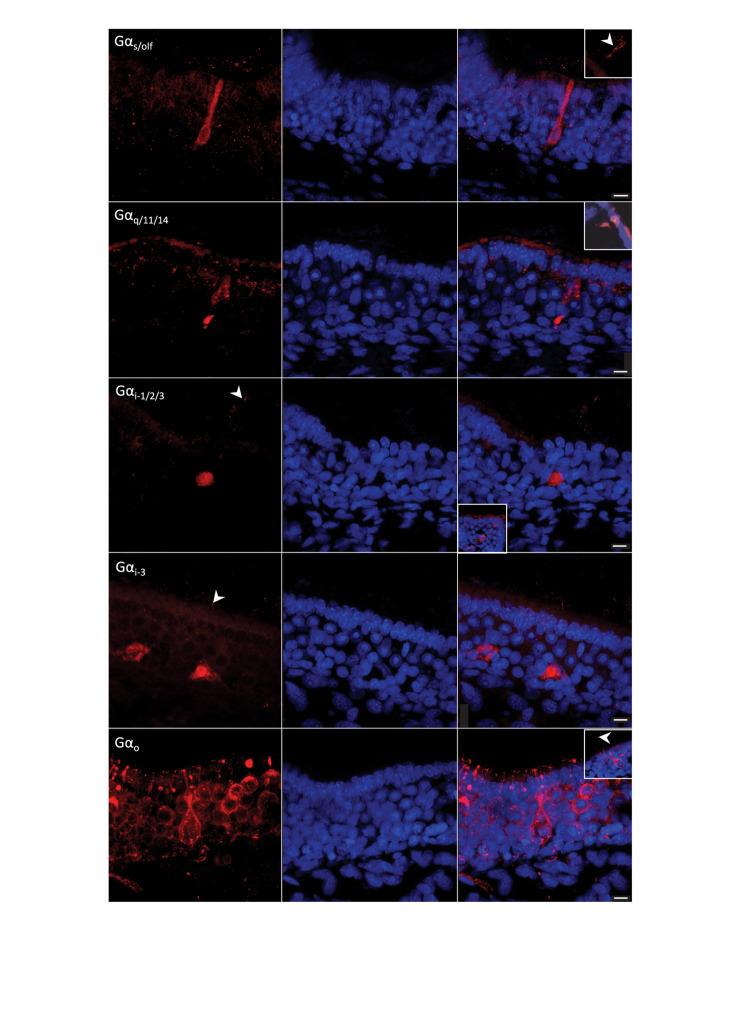
G-protein immunohistochemistry on sections of the olfactory mucosa of *Chiloscyllium punctatum*. The five antisera against G-protein α-subunits used labeled different olfactory receptor neurons (ORNs) (left column, red fluorescence) and Hoechst stained all nuclei (blue). **(First row)** antisera against Gα_s/olf_ labeled mainly long ORNs, which soma sits in the lower layers of the neuroepithelium. These elongated cells have a rounded nucleus occupying most of the soma, a long and relatively narrow dendrite ending in an apical knob, which protrusions were not clearly associated with their soma. This marker also labeled other somas in the intermediate to low layers of the neuroepithelium (sometimes with associated cilia above the neuroepithelium surface but no dendrite clearly labeled), and randomly scattered cilia (sectioned or not). Sectioned labeled cilia showed in inset image. **(Second row)** antisera against Gα_*q/11/14*_ labeled mainly microvillous and crypt ORNs (inset image), and partial labeling of ORN dendrites in the upper neuroepithelium. **(Third row)** antisera against Gα_i–1/2/3_ labeled intermediate somas and possible associated cilia, some more faint apical cells, as well as progenitor cells in the basal layer (inset image). **(Fourth row)** antisera against Gα_i–3_ labeled somas in lower half of the neuroepithelium, possible associated cilia, and ORN axons in the *lamina propria*. **(Fifth row)** antisera against Gα_*o*_ labeled the somas of different microvilli-bearing ORN populations, including the microvillous morphotype, the pear-shaped morphotype with a swelled dendrite, and the teardrop-shaped morphotype, as well as some short (perhaps sectioned) cilia close to the apical membrane, and scarce longer cilia (inset image). White arrowheads, labeled cilia. Scale bars = 10 μm.

Gα_s/olf_ antiserum predominantly labeled long ORNs, i.e., elongated cells, with a soma and a nucleus in the lower part of the SOE and a long dendrite, thinner than the soma, extending to the apical surface (Figure 7). Also labeled by this antiserum in the SOE were some cilia on the apical surface, oval-shaped somas in the basal layer (possible progenitor cells, based on their position, size and shape on histological sections observed under light microscopy, LM – cf. Figure 5B), as well as some circular somas or their upper part of the basal layer (possible immature cells, based on prior LM observations), and some axons in the *lamina propria*. In non-sensory regions, anti-Gα_s/olf_ labeled circular configurations present in goblet cells. Immunoreactivity (–ir) occurred on all cryosections, but did not appear to be prevalent (i.e., could be counted manually across the sections). Generally, Gα_s/olf_ –ir was found on secondary folds rather than in the troughs between folds, and was particularly intense at the posterior part of the lamellae (toward the olfactory bulb), where the SOE is thicker. None of the other antisera tested appeared to label these tall, elongated cells. In all non-chondrichthyan taxa studied to date, the Gα_s/olf_ protein is typical of ciliated ORNs. Here, the presence of Gα_s/olf_ –ir cells in the olfactory epithelium of *C. punctatum* only suggests the presence of ciliated ORNs, as cilia could not be clearly associated with this cell morphotype under scanning or transmission electron microscopy.

Gα_q/__11__/__14_ antiserum labeled two main ORN types in the SOE, but often partially. Gα_q/__11__/__14_–ir was mainly found in intermediate somas and/or thick columnar dendrites (often labeled in a triangular shape), associated with microvilli on the apical surface (i.e., microvillous ORNs). Positive labeling was also detected in rare large, cup-shaped somas, with a large horizontal nucleus at the base of the soma, under the apical surface of the SOE (i.e., crypt-like ORNs) (Figure 7). This antiserum often labeled either the upper or lower parts of intermediate somas (triangular-shaped labeling), or only dendrites and/or cell membranes. Labeled “granules” between the supporting cells (SCs) were also observed in the upper neuroepithelium and were likely to be partially labeled dendrites from microvillous ORNs. Labeling also occurred in oval cells of the basal layer (possible progenitor cells, based on their position, size and shape on prior LM observations – cf. Figure 5B), a rare number of cilia (maybe from crypt cells, although this cannot be confirmed without a corresponding labeled soma), and a limited number of axons. Labeled cells were predominantly found on secondary folds of the SOE. In the non-sensory epithelium, Gα_q/__11__/__14_–ir was concentrated in circular aggregations of vesicles (termed configurations) in goblet cells, as well as in the immature cells in the basal layer.

Gα_i–__1__/__2__/__3_ antiserum labeled intermediate to the basal somas of the SOE, with circular nuclei in the middle (when visible in the section plane) layer, as well as smaller, pear-shaped apical somas, although in smaller numbers and labeled more faintly (Figure 7). Gα_i–__1__/__2__/__3_–ir was also found in some cells of the basal layer, in axons, as well as partially labeled dendrites around SCs. Some cilia were labeled, but most of the labeling was restricted to microvilli. Unknown large and bright round structures were labeled close to the raphe, but these profiles could not be identified as defined cells. In non-sensory regions, a few apical, cup-shaped somas with cilia were labeled, as well as circular configurations in the soma of both immature cells (in the basal layer) and goblet cells (apical layer). Overall, the labeled cells were present in higher numbers at the origin of lamellar single folds near the central raphe, and in between folds.

Gα_i–__3_ antiserum labeled pear-shaped, apical to intermediate somas, with very thin dendrites and apical microvilli (i.e., pear-shaped ORNs), but also labeled lower, teardrop-shaped somas with a basal nucleus, and some apical cilia (i.e., possibly from crypt cells, the somas of which were not labeled) (Figure 7). Some oval somas in the basal layer (possible progenitor cells) and axons in the *lamina propria* were also labeled, as well as sparse granules in the upper SOE (possibly partially labeled dendrites). In non-sensory areas, scarce, large, intermediate somas with wide horizontal nuclei were labeled. Gα_i–__3_–ir was present in higher densities at the periphery of sections, i.e., on the anterior part of the lamellae or at their origin, toward or within the non-sensory epithelium.

Gα_*o*_ antiserum labeled at least three distinct microvilli-bearing cells in the SOE; intermediate columnar somas with vertical, oval nuclei in the middle (microvillous ORNs); intermediate to apical pear-shaped somas with small, circular, basal nuclei and a swelling along their thin dendrite (pear-shaped ORNs); and apical, teardrop-shaped somas with large, round nuclei (teardrop-shaped ORNs). Labeling was also present in rare apical cilia (possibly from crypt-like cells; no somas were labeled but an enlarged, round, non-labeled area was often present underneath these rare cilia), and in axons within the *lamina propria*, in very high proportions within the same section. In the non-sensory areas, goblet cells were not labeled, but some apical, cup-shaped somas with cilia were labeled with anti-Gα_*o*_. Microvillous ORNs were more common near the central raphe. The pear-shaped, swelled ORN type was mainly present on the secondary folds. While teardrop-shaped ORNs were found throughout the epithelium, these cells were present in higher densities within the troughs between secondary folds. Gα_*o*_–ir was highly prevalent (i.e., too dense to allow manual counting) across all sections and appeared to be homogeneously distributed.

#### Non-prevalent G-Protein Immunoreactivity

**FIGURE 8:**
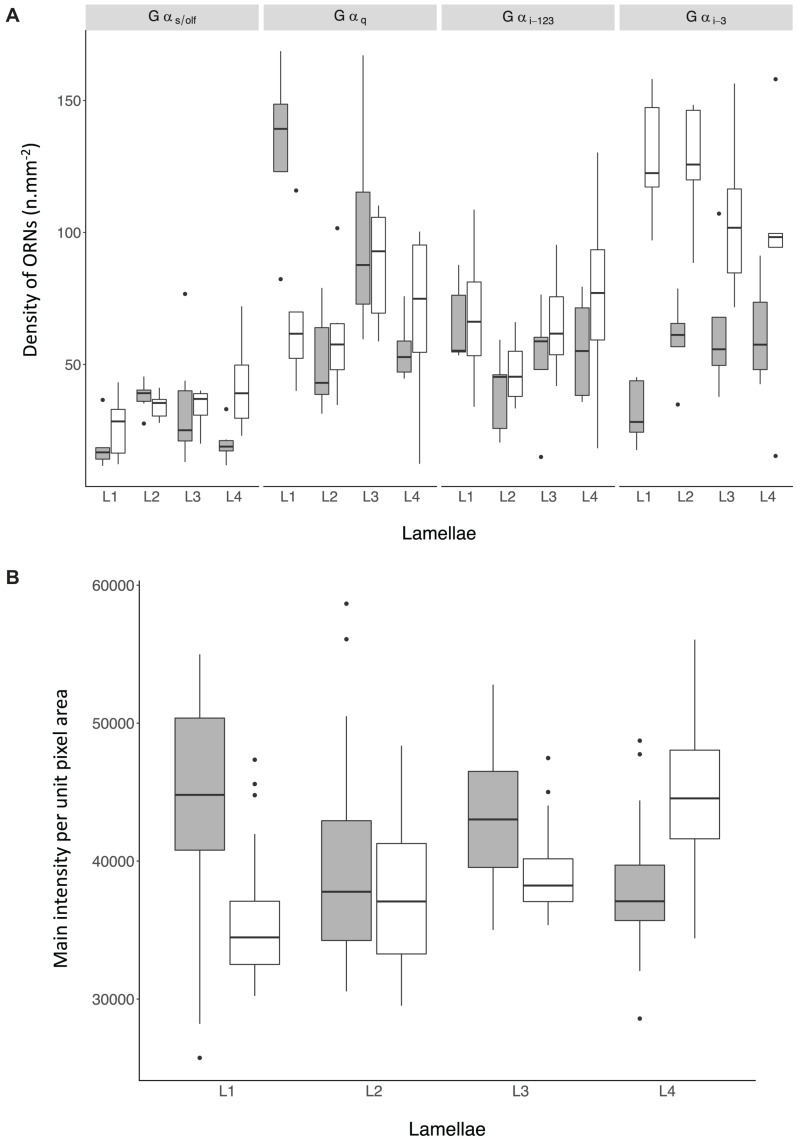
**(A)** Density of non-prevalent Olfactory Receptor Neurons (ORNs) in the olfactory mucosa of *Chiloscyllium punctatum* (CP, *n* = 2) as indicated by the antiserum used to label them (top panels) and the position of the lamellae pairs sampled across the olfactory rosette (L1-2, medial lamellae; L3-4, lateral lamellae). **(B)** Mean pixel intensity per unit area (mm^2^) from prevalent ORNs cells in the olfactory mucosa of *Chiloscyllium punctatum* (CP, *n* = 2) labeled by Gα_*o*_ in function of the position of the lamellae pairs sampled across the olfactory rosette (L1-2, medial; L3-4, lateral). See Figure 1 for reference to positions of lamellae pairs sampled. gray: specimen CP7; white: specimen CP8.

For the four non-prevalent types of anti-G_α_ labeling (i.e., when labeled cells could be counted manually, as defined in “Materials and Methods”), there were no significant differences in densities between lateral (L3, L4 pooled) and medial (L1 and L2 pooled) lamellae, for each treatment (Gα_s/olf_: *df* = 38.69, *t* = −0.28, *p* = 0.78; Gα_q/__11__/__14_: *df* = 37.59, *t* = −0.52, *p* = 0.61; Gα_i–__1__/__2__/__3_: *df* = 33.11, *t* = −0.65, *p* = 0.52; Gα_i–__3_: *df* = 36.44, *t* = 0.35, *p* = 0.73). However, there were significant variations in the mean densities of ORNs labeled between treatments (*n* = 5 antisera used) and specimens (*n* = 2), as well as for the interaction of both terms (*p*-Values < 0.0001) (Figure 8A and Table 3A). The density of ORNs labeled by Gα_s/olf_ was markedly lower than for the other antisera, although all displayed substantial variation between specimens (CP7, 8). Specifically, ORN density labeled by the other Gα antisera was two to three-fold higher than for Gα_s/olf_ with the two specimens pooled (81.1 ± 44.3 SD cell.mm^–2^ for Gα_q/__11__/__14_, 67.2 ± 35.7 cell.mm^–2^ for Gα_i–__1__/__2__/__3_ and 94.1 ± 50 cell.mm^–2^ for Gα_i–__3_ compared to 30.8 ± 13.5 cell.mm^–2^ for Gα_s/olf_). At the specimen level (*n* = 4 lamellae pair pooled), ORN density was, on average, higher in CP8 than CP7 for Gα_i–__1__/__2__/__3_ antiserum (79.9 ± 42.3 cell.mm^–2^ for CP8 versus 52.6 ± 33.4 cell.mm^–2^ for CP7) but significantly higher for Gα_i–__3_ antiserum (two-fold higher: 126.5 ± 45.7 cell.mm^–2^ for CP8 versus 58.1 ± 47 cell.mm^–2^ for CP7). Whereas, ORN densities were relatively similar between specimens for Gα_s/olf_ (34.7 ± 11.5 cell.mm^–2^ for CP8 versus 27.1 ± 14.3 cell.mm^–2^ for CP7) and for Gα_q/__11__/__14_ (77.2 ± 35.2 cell.mm^–2^ for CP8 versus 86.3 ± 54 cell.mm^–2^ for CP7). So, the significant difference between treatments seemed to be mainly driven by Gα_s/olf_ antiserum. No significant differences were found between lamellae (*n* = 4 pairs) or between sections within each lamella (*n* = 6) (Table 3A). Yet, the interaction term between treatment and lamellae was significantly different (*p* = 0.0114).

**TABLE 3A:** Model outputs from the linear regression for the density of non-prevalent labeled Olfactory Receptor Neuron cells in the olfactory mucosa of *Chiloscyllium punctatum* (*n* = 2).

Variable	SS	*df*	*F*-value	*p*-Value
Specimen	11289	1	16.825	<0.001***
Treatment	69168	3	34.361	<0.001***
Lamellae	2843	3	1.412	0.243
Section	5453	5	1.625	0.159
Lamellae/Section	5007	15	0.497	0.937
Specimen:Treatment	26562	3	13.195	<0.001***
Specimen:Lamellae	1461	3	0.725	0.538
Specimen:Section	3369	5	1.004	0.418
Treatment:Lamellae	15258	9	2.526	0.011*
Treatment:Section	6635	15	0.659	0.818

SS, sum of squares; df, degree of freedom; Significant variables *, p < 0.05; **, p < 0.005; ***, p < 0.001.

For the interaction between treatment and specimen, i.e., comparing the same treatment between specimens or different treatments within the same specimen (lamellae and sections pooled), *post hoc* Tukey tests showed that densities significantly differed within specimens predominantly, rather than between specimens, except for treatment Gα_i–__3_ (*p* < 0.0001) (Supplementary Figure 7 and Supplementary Table 8). For specimen CP7, cell densities were significantly different between all treatments (all *p*-Values < 0.005), except between treatments Gα_i–__1__/__2__/__3_ and Gα_i–__3_ (*p* = 0.9223). As for specimen CP8, cell densities significantly differed between all treatments (all *p*-Values < 0.0005), except between treatments Gα_q/__11__/__14_ and Gα_i–__1__/__23_ (*p* = 0.6017) (Supplementary Figure 7).

When looking at the interaction between treatments and lamellae pairs (i.e., comparing the same treatment between lamellae or different treatments within the same lamellae pair, specimens pooled), there were significant differences between treatments within lamella, rather than for the same treatment between lamellae, except for Gα_q/__11__/__14_ (Figure 15; highlighted in Supplementary Figure 7). The density of cells labeled by Gα_q/__11__/__14_ significantly differed between all four lamella pairs (all *p*-Values < 0.05); 98.7 ± 50.2 SD cell.mm^–2^ for L1, 60.1 ± 28.8 cell.mm^–2^ for L2, 106.6 ± 62.8 cell.mm^–2^ for L3, and 70.2 ± 30.9 cell.mm^2^ for L4. Across lamella pairs under each treatment, specifically, lamella pairs L1 (most medial) and L4 (most lateral) were driving the variation in cell density labeled with Gα_s/olf_ (Gα_s/olf_ versus Gα_q/__11__/__14_, Gα_s/olf_ versus Gα_i–__1__/__2__/__3_ and Gα_s/olf_ versus Gα_i–__3_; all *p*-Values < 0.0005 and <0.005, for L1 and L4 respectively). While, lamella pair L2 showed significant differences between only two treatment comparisons (Gα_i–__1__/__2__/__3_ versus Gα_q/__11__/__14_ and Gα_i–__1__/__2__/__3_ versus Gα_i–__3_; both *p*-Values < 0.005). The density of labeled cells in lamella pair L3 was significantly different across all treatment combinations (all *p*-Values < 0.005).

#### Prevalent G-Protein Immunoreactivity

For the prevalent labeling observed from Gα_*o*_ (i.e., when labeled cells could not be counted manually, and the mean intensity of labeling per unit pixel area was used as a proxy for labeled cell density, as defined in “Materials and Methods” section “Immunohistochemistry and Confocal Microscopy”), the mean intensity per unit pixel area was similar between specimens (lamellae and sections pooled: 40,952.07 ± 6687.62 for CP7; 39,921.58 ± 5769.47 for CP8; *p* = 0.09) (Figure 8B and Table 3B). However, we found that the mean intensity was significantly different between lamellae (*p* < 0.0001). In particular, the mean intensity was significantly higher in lateral lamellae (specimen and lamellae L3-4 pooled: 82,841.88 ± 5,185.07) than medial lamellae (specimen and lamellae L1-2 pooled: 78,456.92 ± 6,987.63) (Gα_*o*_: *n* = 2, *df* = 218.12, *t* = −2.18, *p* = 0.03). Significant differences in mean intensity were also found between sections, within lamellae (*p* < 0.0001), with anterior sections showing relatively higher mean intensities (s1, 43,967.18 ± 5,749.73; s2, 39,622.08 ± 6,722.17; s3, 40,118.13 ± 6,523.68) than posterior ones (s4, 39,089.43 ± 5,522.88; s5, 38,442.91 ± 5,797.55; s6, 40,085.58 ± 3,740.02). Interaction terms between lamellae and section, specimen and lamellae, and specimen and section were also significantly different (all *p*-Values < 0.0001).

**TABLE 3B:** Model outputs from the linear regression run on the density of prevalent labeled Olfactory Receptor Neuron cells in the olfactory mucosa of *Chiloscyllium punctatum* (*n* = 2).

Variable	SS	*df*	*F*-value	*p*-Value
Specimen	6.2864E + 14	1	38.929	0.099
Lamellae	1.9336E + 14	3	3.991	<0.001***
Section	8.4995E + 14	5	10.526	<0.001***
Lamellae/Section	1.5718E + 15	15	6.489	<0.001***
Specimen:Lamellae	2.0265E + 15	3	41.831	<0.001***
Specimen:Section	2.2370E + 14	5	2.770	<0.001***

SS, sum of squares; df, degree of freedom; Significant variables *, p < 0.05; **, p < 0.005; ***, p < 0.001.

*Post hoc* Tukey tests further revealed significant relationships between some biologically relevant contrasts, but not all (Supplementary Figure 9 and Supplementary Table 10). For the interaction between lamellae and sections (specimens pooled, *n* = 2) particularly, the significant differences in mean intensity found were inconsistent between the four lamellae pairs (*n* = 4), and mainly driven by intra-lamellar differences for lamellae pairs L1 and L2, with most differences in section pairwise comparisons (*n* = 7–9) arising from sections “Introduction” and “Materials and Methods” (most anterior sections) compared to all other sections (all *p*-Values < 0.004 for L1; < 0.008 for L2). While, other significant intra-lamellar differences in mean intensity were also arising mainly from section “Materials and Methods” in L3 (all *p*-Values < 0.03), and from section “Introduction” in L4 (all *p*-Values < 0.04).

For the interaction between specimen and lamellae (sections pooled, *n* = 6), most of the variation stemmed from significant differences in mean intensity between specimens (for most lamellae pairs, i.e., L1, L3, and L4; all *p*-Values < 0.002) and between lamellae within specimen, especially due to lamellae pair L4 (most lateral) in both specimens (all *p*-Values < 0.0001). The interaction between specimen and section (lamellae pair pooled, *n* = 4) was less important for the scope of this study, because we aimed to test for differences in the distribution of labeled cells across the olfactory organ (i.e., between and within lamellae). However, results still indicate and emphasize that most anterior sections (“Introduction” and “Materials and Methods”) had significantly higher mean intensity values per unit pixel area than other, more posterior sections (“Results,” “Discussion,” “Figures,” and “Tables”) for both specimens (all *p*-Values < 0.04), specifically driven by section “Introduction” in CP7 (see values in paragraph above, and Supplementary Table 10).

### Hydrodynamic Simulations in the Olfactory Cavities

**FIGURE 9:**
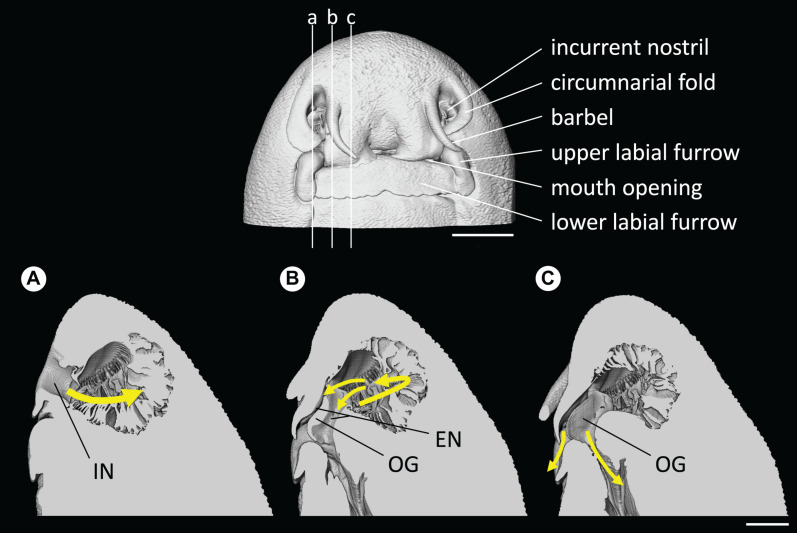
Head and nostril morphology of *Chiloscyllium punctatum*, presented from the volume data (CT). **(Top)** the vertical lines indicate the position of sagittal slices presented in panels **(A–C)**. **(Bottom)** main water flow pathway in the olfactory cavity, represented by gradient, colored arrows **(a–c)**. IN, incurrent nostril; EN, excurrent nostril; OG, oronasal groove. Scale bars = 5 mm.

Water flow was channeled by the circumnarial fold into the incurrent nostril and was directed into the olfactory cavity, while a small portion of the incoming flow was deflected by the minor nasal fold at the entrance of the incurrent nostril creating a localized vortex. Once in the cavity, water was then circulated from the lateral to the medial region of the olfactory cavity, along the central raphe of the olfactory rosette, which is the incurrent channel. The flow is likely diffusing between single lamellar folds, although only the global fluid dynamics, at the scale of the overall cavity, were examined in this study. At the lateral end of the cavity, the flow is directed toward the excurrent nostril, which is divided into two channels separated by the oronasal groove, underneath the labial flap (Figure 9; and see Supplementary Video 2). One excurrent channel connects with the buccal cavity in the mouth and the other excurrent channel connects with the exterior between the labial flap and the oronasal groove.

**FIGURE 10:**
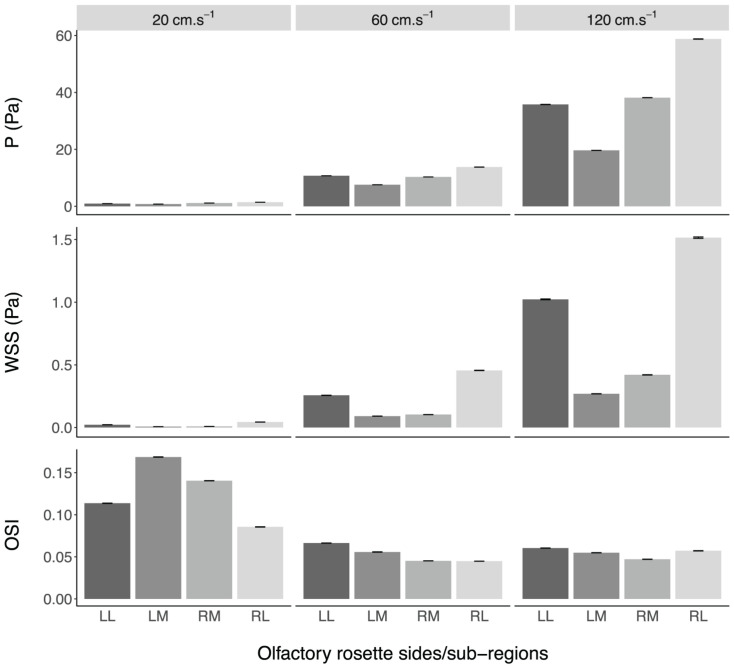
Mean values ± standard error of the converged pressure (P) and wall shear stress (WSS), and spontaneous oscillatory shear index (OSI), in the olfactory cavities of *Chiloscyllium punctatum* (*n* = 1) per olfactory rosette side and sub-region, at three different fluid speeds. LL, left olfactory cavity, lateral sub-region; LM, left olfactory cavity, medial sub-region; RM, right olfactory cavity, medial sub-region; RL, right olfactory cavity, lateral sub-region.

**FIGURE 11:**
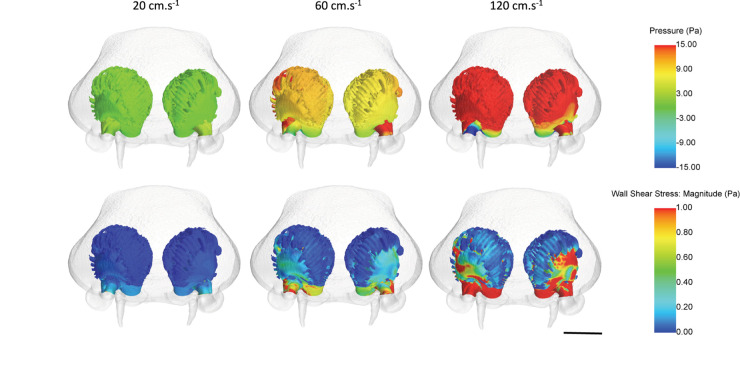
Mean pressure and wall shear stress values across the olfactory cavities of *Chiloscyllium punctatum* (in frontal view) under three inlet flow velocities, from converged simulations. Scale bar = 5 mm. Individual figures created in STAR-CCM + (v12.06, Siemens, Berlin).

Fluid dynamic simulations show that mean converged pressure (P) and wall shear stress (WSS) values are significantly higher at increased flow velocities (all *p*-Values < 0.0001) (Figures 10, 11). Conversely, spontaneous oscillatory shear index (OSI) is significantly lower at swimming speeds of 120, rather than 20 cm.s^–1^ (*p* < 0.0001). However, mean spontaneous OSI values are relatively similar at speeds between 60 and 120 cm.s^–1^. For all parameters (P, WSS and spontaneous OSI), *post hoc* Tukey tests on the significant interaction between sides and sub-regions indicates significant contrast terms (*p* < 0.0001) for all biologically relevant pairwise comparisons (i.e., within the same side, left or right rosette). For the two converged parameters (P and WSS), the mean values are significantly higher in the right versus the left olfactory cavity, and for the lateral versus the medial regions of the cavities (both sides) (all *p*-Values < 0.0001), whereas, mean spontaneous OSI values were higher in the left cavity versus the right cavity (*p* < 0.0001) across all fluid velocities. At the lowest flow velocity, OSI was significantly higher in the medial rather than the lateral regions, but opposite under the higher flow velocities (all *p*-Values < 0.0001).

## Discussion

This study examined several morphological aspects of the ultrastructural organization of the olfactory rosette in the brownbanded bamboo shark *Chiloscyllium punctatum* and sought to increase our knowledge of possible functional adaptations of the peripheral olfactory nervous system in an elasmobranch model species, using a multimodal approach.

### Gross Morphology of the Olfactory System

Investigation of the olfactory system of *C. punctatum* revealed that, as in other elasmobranch species, such as the small eye hammerhead *Sphyrna tudes* (Rygg et al., 2013), water enters the incurrent nostril (nare) on either side of the head, which opens onto an olfactory organ (rosette), comprised of many primary folds (lamellae), and circulates from lateral to medial regions of the olfactory cavity. The fluid dynamic simulations showed that two excurrent paths exist in *C. punctatum*: one through the mouth and the other through the space between the labial flap and the oronasal groove. The connection between the excurrent nostril and the mouth in this species is characteristic of “Group B,” as proposed by Cox (2013). Although assessment across a broader range of species is required, this appears to be a morphological difference in the olfactory cavity between sedentary species (i.e., most benthic species), such as *C. punctatum*, and active swimming species, such as the lemon shark *Negaprion brevirostris* and the silky shark *Carcharhinus falciformis* (Zeiske et al., 1987; Cox, 2013). This difference could reflect an adaptation to sample chemical cues (both olfactory and gustatory) more efficiently in sedentary species. From the main incurrent flow, water disperses into the inter-lamellar space as it passes along the central raphe of the olfactory rosette.

The olfactory cavity is filled with an olfactory organ (rosette), comprised of many primary folds (lamellae). Using scanning electron microscopy, we showed that the olfactory lamellae of *C. punctatum* are composed of two lamella folds, which are almost separated (type II; Cox, 2013) as shown in Figure 1, and mostly covered by neuroepithelium (sensory olfactory epithelium, SOE), as already revealed in this species (Schluessel et al., 2008). The distribution of neuroepithelium has been found to be patchier in some benthic elasmobranchs, such as the Port Jackson shark *Heterodontus portusjacksoni*, where inlets of non-sensory epithelium can be found within the neuroepithelium, and *vice versa* (Schluessel et al., 2008). A maximized neuroepithelial surface area may be another olfactory specialization. However, the overall epithelial organization and cell ultrastructure appears to be conserved across species; for example, the types of cells found in the two epithelia, i.e., supporting cells in the neuroepithelium and non-sensory cells in the non-sensory epithelium, showed similarities with other elasmobranch species studied to date (Zeiske et al., 1987; Schluessel et al., 2008; Theiss et al., 2009).

Following detection of biologically relevant chemicals by olfactory receptor neurons (ORNs) in the neuroepithelium, olfactory signals are conveyed by ORN axons (primary projections) to the olfactory bulbs. DiceCT imaging enabled us to reveal a lateral partitioning of primary projections to the olfactory bulb, as well as a compartmentalized olfactory bulb internally, comprising two clusters of glomeruli matching the two external swellings visible at the olfactory bulb surface in *C. punctatum*. Specifically, nerve bundles emanating from lamellae project toward the corresponding “sub-bulb,” whereby the lateral lamellae project to the lateral cluster of glomeruli and the medial lamellae project to the medial glomerular cluster in the olfactory bulb. This is similar to the projection patterns described in the Atlantic sharpnose shark *Rhizoprionodon terranovae*, the bonnethead shark *Sphyrna tiburo* (Dryer and Graziadei, 1993), the lemon shark *Negaprion brevirostris*, and the Atlantic stingray *Hypanus sabina* (Meredith et al., 2013). This anatomical segregation may indicate a somatotopic organization (based on location) of the primary projections in the olfactory bulb of *C. punctatum* (Daniel, 1934; Dryer and Graziadei, 1993, 1994, 1996; Meredith et al., 2013), as opposed to a topographic (based on function) organization widely recognized in teleost fishes (Hara and Zhang, 1996; Friedrich and Korsching, 1998; Morita and Finger, 1998; Laberge and Hara, 2001; Nikonov and Caprio, 2001; Hansen et al., 2003), only proposed for one elasmobranch to date, the small spotted catshark, *Scyliorhinus canicula* (Ferrando et al., 2009). Although our 3D observations do not provide a definitive assessment of such organization, the characterization of ORN types, their distribution across the olfactory rosette, and their function in detecting specific classes of odorants would help to clarify how information is mapped into the elasmobranch olfactory bulb.

### Olfactory Receptor Neuron Types

We identified at least five distinct morphological types of ORNs in this species, based on their soma size, shape and position within the neuroepithelium, the nucleus size, shape and position within the soma, the appearance of their apical dendrites, as well as of the apical knob, using light and electron microscopy combined with G-protein immunohistochemistry (IHC). The different morphotypes identified in *C. punctatum* tend to resemble the five types more recently described in teleosts (i.e., ciliated, microvillous, crypt, Kappe and pear ORNs) (Zeiske et al., 1992; Morita et al., 1996; Hansen and Zielinski, 2005; Saraiva and Korsching, 2007; Wakisaka et al., 2017; Calvo-Ochoa and Byrd-Jacobs, 2019); although, this will require further investigation, including molecular characterization. To date, only microvillous and crypt ORNs have been previously described in elasmobranchs, other than *C. punctatum* (Theisen et al., 1986; Takami et al., 1994; Ferrando et al., 2006a, 2007; Schluessel et al., 2008; Theiss et al., 2009; Cox, 2013; Quintana-Urzainqui et al., 2014).

We reveal the presence of tall, elongated ORNs, which display Gα_s/olf_ immunoreactivity (–ir). Our SEM and TEM observations do not allow us to confidently associate a ciliated apical knob to this cell morphotype although the presence of rare rootlets beneath the apical knob could suggest it. This would confirm previous findings about the absence of this cell type in the class Chondrichthyes. Nonetheless, based on the positive Gα_s/olf_ –ir observed, we provide at least the evidence of another ORN type in this species, potentially resembling the ciliated type described in other vertebrates. Indeed, based on the shape and position in the neuroepithelium (long dendrite, round soma and nucleus in the intermediate to basal layers of the SOE), the morphological characteristics of the “long” or “elongated” cell morphotype observed resemble those of cell types found in other vertebrate groups; for example, the ciliated ORNs described in the olfactory epithelium of teleost fishes (where the cell spans the entire neuroepithelium, has a round and basal soma, a long and thin dendrite, the presence of cilia rootlets and/or a ciliated knob), such as the goldfish *Carassius auratus* (Hansen et al., 2003, 2004), the Senegal or gray bichir *Polypterus senegalus* (Ferrando et al., 2011) and the zebrafish *Danio rerio* (Biechl et al., 2017). It could also resemble the “tall” ORNs described in agnathans, such as the sea lamprey *Petromyzon marinus*, at its metamorphic stage (Laframboise et al., 2007). The long cell type described in this study also shows morphological similarities with the ciliated ORNs in the vomeronasal epithelium of anurans and salamanders or microvillar ORNs in the vomeronasal epithelium of mammals (Eisthen, 1992, 1997). Since only short or part of (i.e., sectioned) cilia rootlets were found underneath the apical membrane (rather than surface cilia) of these tall, elongated cells, using TEM (Figure 6A), it is not possible to definitively characterize these ORNs as ciliated. No ciliated ORNs have been morphologically identified in any chondrichthyan (using light or electron microscopy), and no Gα_s/olf_ –immunoreactive cells have been reported in the chondrichthyan species studied to date; i.e., in the small-spotted catshark *Scyliorhinus canicula* (Ferrando et al., 2006a), the thornback ray *Raja clavata* (Ferrando, 2008), and the Greenland shark *Somniosus microcephalus* (Ferrando et al., 2016). Therefore, our description of Gα_s/olf_–immunoreactive cells can only be putative. As this is a novel result, it will require further morphological investigation, combined with molecular characterization of the receptor molecule associated with this ORN morphotype and G-protein immunoreactivity, preferably in mature individuals.

Microvillous ORNs were identified by a shorter and thicker cell morphotype, with an intermediate soma position within the SOE, and a dendrite slightly thinner than the medial/basal soma, giving them a more columnar appearance. This cell type was labeled by Gα_q/__11__/__14_, Gα_i–__1__/__2__/__3_ and Gα_*o*_ in this species, reflecting similar IHC results to *C. auratus* (labeled by Gα_q/__11__/__14_ Gα_i–__3_ and Gα_*o*_) (Hansen et al., 2004). Previous studies in other cartilaginous fishes, including the shark *S. canicula* (Ferrando et al., 2009) and the rabbit fish *Chimaera monstrosa* (Ferrando et al., 2010), have revealed that microvillous ORNs have been only labeled by Gα_*o*_; while, this ORN morphotype has been labeled with both Gα_i–__3_ and Gα_*o*_ in the Greenland shark *Somniosus microcephalus* (Ferrando et al., 2016).

Rare crypt-like cells were also identified, with a larger, cup-shaped soma in the upper layer of the neuroepithelium, a large, horizontal ovoid nucleus, and an apical knob sitting at the bottom of a depression. In *C. punctatum*, the soma of crypt-like ORNs were labeled with Gα_q/__11__/__14_, which is similar to IHC results described in *C. auratus* (Hansen et al., 2004). However, rare apical knobs bearing cilia were labeled with Gα_i–__1__/__2__/__3_ and Gα_i–__3_ in our study, versus Gα_*o*_ in *C. auratus* (Hansen et al., 2004). Cilia were not directly observed under light and electron microscopy, but the rare cilia labeled were often associated with positive labeling within the neuroepithelium underneath; whether the associated labeling was from this cell morphotype could not be distinguished. The unique morphology of this cell type allowed us to confirm its presence using light microscopy and IHC, although, their rare occurrence and visualization of partial profiles on sections (particularly regarding the lack of cilia observed) prevented us from describing their ultrastructure using TEM.

At least three other microvilli-bearing cell morphotypes were observed. Pear-shaped ORNs were labeled with anti-Gα_i–__1__/__2__/__3_, Gα_i–__3_ and Gα_*o*_ in *C. punctatum*, while Biechl et al. (2017) found cells with a similar morphotype associated with Gα_s/olf_ in zebrafish *Danio rerio*. Kappe-like ORNs were identified histologically in *C. punctatum*, in our study, but were not clearly labeled using IHC. As this ORN type has been associated with Gα_*o*_ in *D. rerio*, a G-protein marker that labeled most of the different cell populations in our study, it is possible that their presence was missed due to a similar soma position as the pear-shaped cells in the neuroepithelium, for instance. Particularly, this could be the case if their abundance was low compared to other microvilli-bearing ORN types. It is worth noting that a teardrop-shaped cell morphology was also observed. These cells were labeled only by anti-Gα_*o*_, and appeared to have a distinctive shape, compared to the pear-shaped cells described previously in histological sections (using both LM and TEM). These teardrop-shaped cells have a soma located within intermediate to low layers of the neuroepithelium, usually shallower than pear-shaped somas, a thin dendrite with a consistent swelling on its apical end, and an apical knob bearing microvilli. This teardrop-shape cell type with a dendritic swelling has not been previously described in any fish species to our knowledge. While this study provides evidence for the presence of more than two ORN types in *C. punctatum*, molecular characterization of the receptors associated with the G-protein expressed for each cell morphotype identified would be critical to confirm the existence of such ORN diversity in this species, and will help us in teasing out the number of receptor molecules present, particularly given the increasing availability of genome sequences for this fish group (including *C. punctatum*) (Chen et al., 2014; Venkatesh et al., 2014; Read et al., 2017; Marra et al., 2019).

Each antiserum labeled small, oval cells in the basal layer of the neuroepithelium, just above the *lamina propria*, in varying numbers depending on the antiserum. Based on their position, size and shape on prior light microscopy observations where we could identify them (cf. Figure 5B), we suggest that the positive labeling of these basal cells could be “possible progenitor cells” and refer to them as such. Future research could include double-staining experiments, using specific markers for progenitor cells, to identify such cells more definitively.

### Abundance and Differential Distribution of ORN Populations

G-protein immunohistochemistry not only allowed us to assess the ORN morphotypes, but also ORN density, as well as small- and large-scale distribution patterns. This study confirms a certain degree of partitioning in cell types present across the rosette and a level of morphological compartmentalization in the olfactory bulbs, a finding which may indicate some functional organization of primary olfactory projections. On lamellar sections, our results showed small-scale differential distribution of the different ORN populations across the rosette, similar to that found in *C. auratus* (Hansen et al., 2004), although the specific patterns differed slightly. For instance, Gα_s/olf_ –ir, which only labeled ciliated ORNs, was found in all sections but predominantly on secondary folds and in higher proportions on posterior parts of lamellae toward the olfactory bulb. Gα_*q*_–ir was evident across the whole of the sensory olfactory epithelium, particularly on the secondary folds, unlike in *C. auratus*, which does not possess secondary folds (Hansen et al., 2004). Gα_i–__1__/__2__/__3_ –ir was more pronounced in troughs (between secondary folds) and generally more common at the origin of lamellar folds (i.e., close to the central raphe). Gα_i–__3_–ir was intense in peripheral regions close to the non-sensory epithelium (both close to the raphe and to the distal edge of the fold), which shows similarities (peri-raphe) with *C. auratus* (Hansen et al., 2004). Gα_*o*_–ir was the most prevalent labeling and labeled three microvilli-bearing ORNs in high proportions throughout the epithelium. The teardrop-shaped ORNs labeled with Gα_*o*_ were more abundant on the secondary folds, whereas the pear ORNs were more common in troughs, with microvillous ORNs more numerous close to the raphe.

On a larger scale, across the olfactory rosette, both the prevalent (Gα_*o*_) and less prevalent Gα–ir (Gα_s/olf_, Gα_q/__11__/__14_, Gα_i–__1__/__2__/__3_, Gα_i–__3_) showed some degree of differential distribution of ORN populations, from most medial lamella pairs (L1) to most lateral lamella pairs (L4). Overall, there were two spatial distributions amongst treatments (antisera used): (1) those which showed different densities of labeled cells between inner (L2 and L3) and outer (L1 and L4) lamella pairs (Gα_s/olf_, Gα_i–__3_ and Gα_*o*_), and (2) those which displayed different densities in alternating lamellae; for example, higher densities in L1 and L3 and lower densities in L2 and L4 (for Gα_q/__11__/__14_ and Gα_i–__1__/__2__/__3_). Statistically, Gα_*o*_ was the only treatment (antiserum) which showed a heterogeneous labeling between lateral and medial regions of the olfactory rosette, as well as intra-lamellar variations between anterior and posterior regions (from s1 to s6). Specifically, Gα_*o*_-labeled ORNs were most abundant in lateral lamellae, and in anterior parts of the lamellae in general. Although Hansen et al. (2004) also reported heterogeneous distribution of Gα_*o*_–ir ORNs in *C. auratus*, the differential distribution described was within, not between, lamellae. In their study, microvillous-type ORNs labeled by Gα_*o*_ were more abundant in dorsomedial regions of the lamellae, close to, and on the middle raphe. Based on the labeling of the different populations of cells in the present study, the microvilli-bearing ORN types (i.e., microvillous, pear, teardrop-shaped with swelling) were the most commonly labeled, two of which (microvillous and pear) were labeled across three treatments. Whereas, ciliated ORNs were less common throughout the neuroepithelium and crypt ORNs were only rare. The differential distributions highlighted in the study suggest a level of anatomical organization in the way water is sampled by the olfactory organ in this species. Future research should aim to ascertain whether this may be a common occurrence across elasmobranch species. Above all, determining which classes of chemicals are detected by each ORN type, or knowing which receptor molecules are expressed on each sub-type of the same ORN population, would be fundamental prior to further interpretation.

### Fluid Dynamic Simulations and Their Functional Implications

For all hydrodynamic parameters (pressure, wall shear stress and spontaneous oscillatory shear index), values were higher in the lateral versus the medial region of the olfactory cavities, apart from a spontaneous oscillatory shear index at the lowest velocity. As the spontaneous oscillatory shear index represents the temporal fluctuation of wall shear stress (localized movement of particles against the surface), this index tends to be higher at low flow velocities, where particles are moving in all directions. Overall, we observed that flow dynamic parameters were higher on the lateral than the medial region, under the three flow velocities (i.e., simulated swimming speeds), and that these lateral differences increased with flow velocity. A comparable gradient in pressure was found across the olfactory organ (from lateral to medial) in the hammerhead shark *S. tudes*, at a similar maximum simulated swimming speed of 155 cm.s^–1^ (Rygg et al., 2013). Specifically, the flow pressure was shown to decrease from 50 to 10 Pa between lateral and medial regions of the olfactory cavity in *S. tudes*, and from 40 to 10 Pa in *C. punctatum*, despite marked differences in the nostril position between species. In *C. punctatum*, the nostrils are positioned on the ventral side of the snout, facing downwards (Supplementary Video 2), and water exits through the mouth and the gills (Figure 9C). The anatomy is different in *Sphyrna spp.*, where both inhalant and exhalant nostrils are positioned on the lateral side of the snout and face forwards (Abel et al., 2010; Rygg et al., 2013). Despite similar pressure gradients (under comparable simulated swimming speeds) between these two species, the extent to which morphological variations (in the olfactory lamellae, rosette, cavity and openings across species; Cox, 2013) influence the hydrodynamic properties in the olfactory cavity requires further investigation. Although we noticed differences between left and right rosettes, this is likely due to sample fixation and preservation, as well as digital corrections made to re-establish the symmetry of the barbels and nares in the individual *C. punctatum* used. The broad internal hydrodynamics of the olfactory cavity may explain the strategic importance of differential distribution in ORNs in sampling classes of odourants more effectively.

### The Role of Olfaction in Shark Behavior

A multimodal approach enabled us to correlate ultrastructural aspects of the olfactory organ to the functional organization of the olfactory cavity and subsequent incurrent flow dynamics. In teleost fishes, feeding and social information are encoded by different types of ORNs. Although many studies reveal variation in the functional differentiation of ORN types between species, typically feeding cues (e.g., amino acids, nucleotides) are found to be mediated predominantly by microvillous ORNs (Speca et al., 1999; Sato and Suzuki, 2001; Hansen et al., 2003; Schmachtenberg and Bacigalupo, 2004; DeMaria et al., 2013), as well as ciliated (Sato and Suzuki, 2001; Hansen et al., 2003; Schmachtenberg and Bacigalupo, 2004; Sato and Sorensen, 2018) and crypt ORNs to a lesser degree (Vielma et al., 2008; Bazáes and Schmachtenberg, 2012). In contrast, social cues (e.g., pheromones for reproduction, and bile salts for social communication) are mediated by either ciliated or microvillous ORNs (for pheromones) (Zippel et al., 1997; Yabuki et al., 2016; Sato and Sorensen, 2018), and ciliated or crypt ORNs (for bile salts) (Vielma et al., 2008; Døving et al., 2011; Bazáes and Schmachtenberg, 2012).

Therefore, based on existing knowledge for teleosts, the higher density of microvilli-bearing ORNs found within the lateral region of the rosette in *C. punctatum* may mediate the detection of certain chemicals more efficiently. Whether this is a functional adaptation to detect predominantly feeding cues versus social cues in the lateral versus medial sides of the rosette in relation to water flow dynamics remains to be tested. This is because the type of information each ORN type encodes in this elasmobranch species, or any other species of this fish group, is currently unknown, and is likely to be species-specific (or at least genus-specific), as found in teleosts. Indeed, previous studies show that given ORN morphotypes can detect various classes of chemicals (e.g., amino acids, bile salts, prostaglandins) depending on the species in teleosts (Zippel et al., 1997; Speca et al., 1999; Sato and Suzuki, 2001; Hansen et al., 2003; Schmachtenberg and Bacigalupo, 2004; Vielma et al., 2008; Døving et al., 2011; Bazáes and Schmachtenberg, 2012; DeMaria et al., 2013; Yabuki et al., 2016). The higher flow pressure in the lateral part of the olfactory cavity may favor the detection of more abundant, overlapping, or heterogeneous cues by more “generalist” receptors, such as those mediating feeding cues. While, the lower flow pressure in the medial part of the cavity may favor the detection of other less abundant, more specific ligands, such as pheromones and/or other social cues. Following the “different tuning hypothesis” referred to in Ferrando and Gallus (2013), one could assume that microvilli-bearing ORN morphotypes may be more broadly versus finely tuned ORN sub-types, given their higher prevalence in the lateral part of the rosette, i.e., higher flow pressure region of the cavity. However, this assumption would need to be experimentally tested in elasmobranchs. Future work should include electrophysiological recording of the responses from ORNs in different regions of the olfactory epithelium and their terminals within each of the sub-olfactory bulb regions during exposure to several types of molecules/odors, to determine whether there is any level of functional organization. Retrograde tracing of the ORNs, as conducted in teleosts (Hansen et al., 2003), would also help clarify the terminal projection patterns within the two hemi-bulbs.

Here, the identification of at least five ORN morphotypes in *C. punctatum* contrasts with previous studies, which characterized one ORN morphotype in the neuroepithelium of *S. microcephalus*, for instance, as reported by Ferrando et al. (2016). This may reflect possible interspecific differences in the role that olfaction plays in the ecology of different species. *C. punctatum* is a small, benthic predatory shark, which inhabits a range of subtidal, intertidal and reef-associated habitats (i.e., a highly dynamic environment), from subtropical to tropical regions of the Indo-Pacific (White and Potter, 2004; Last and Stevens, 2009; Chin et al., 2012; Akhilesh et al., 2014). Members of this family possess average to small olfactory bulbs (relative to the whole brain), as compared to a wide range of elasmobranchs (Yopak et al., 2015). This species uses suction feeding to capture small, benthic invertebrates (e.g., annelids and crustaceans) and fishes (Harahush et al., 2007; Lowry and Motta, 2007; Last and Stevens, 2009; Chin et al., 2012), which may require locating cryptic, mobile prey quickly, and tracking them in complex habitats. In comparison, species such as *S. microcephalus* or the tiger shark *Galeocerdo cuvier* display relative large olfactory bulb size and are known to be rather migratory and scavengers. But whether interspecific differences in the presence (or lack thereof) of multiple ORN types can be attributed to phylogeny or to ecological and behavioral parameters (i.e., similar to olfactory bulb size) requires further investigation. Future studies should prioritize the molecular characterization of ORN types present across members from different orders and/or families to assess/confirm whether such adaptations exist within the neuroepithelial ORN populations in elasmobranchs, before aiming to ascertain whether these potential differences correlate with phylogeny versus ecological factors such as habitat and/or lifestyle.

This is the first study to use an integrated approach to morphologically and immunohistochemically characterize ORN types in *C. punctatum* and their differential distribution, in combination with fluid dynamics, to trace water inflow into the olfactory cavity. Together, these results show that the high densities of microvilli-bearing ORNs within the lateral region of the rosette and anterior parts of the lamellae are important for sampling incoming odorants during swimming and may determine subsequent tracking behavior in *C. punctatum*. Whether the differential distribution of ORNs and the lateral partitioning of glomerular structures in the olfactory bulbs is related to olfactory function, i.e., processing and segregating inputs with respect to feeding and social interactions, remain to be tested.

## Data Availability Statement

The raw data supporting the conclusions of this article will be made available by the authors, without undue reservation.

## Ethics Statement

The animal study was reviewed and approved by the University of Western Australia (UWA) Animal Ethics Approval No. RA/3/100/1153.

## Author Contributions

VC-A, JS, BD, JP, KY, and SC conceived the study. VC-A designed the data collection and analyses, conducted the research (assisted by AH under the technical guidance of PR for the immunohistochemistry, and assisted by HC under the technical guidance of BD for the hydrodynamics), performed the statistical analyses using the custom-written script by AM to extract the information from the raw immunohistochemically labeled images, created the figures and tables, and wrote the draft of the manuscript. HC ran the 3D simulations of the water flow dynamics applied to the shark’s head surface model prepared by VC-A and JS, provided the raw data to VC-A for statistical analysis, and created Figure 2, Supplementary Figure 3, Supplementary Tables 4, 5, and Supplementary Video 2. HC wrote the “Computational Fluid Dynamics” section of this manuscript, except the “Surface Mesh Generation” section, which was written by JS. All authors contributed to the manuscript revision, read, and approved the submitted version.

## Conflict of Interest

The authors declare that the research was conducted in the absence of any commercial or financial relationships that could be construed as a potential conflict of interest.
