# Supplementary material for: Multimodal Imaging and Analysis of the Neuroanatomical Organization of the Primary Olfactory Inputs in the Brownbanded Bamboo Shark, Chiloscyllium punctatum
Source: Front Neuroanat. 2020 Nov 26;14:560534. doi: 10.3389/fnana.2020.560534 (PMC7726474; doi:10.3389/fnana.2020.560534)
Supplement: Supplementary file 3 [file Data_Sheet_1.pdf]

Supplementary Material

Supplementary Material S1. Script developed and used in Fiji (ImageJ 1.52n, NIH, USA) to assess cell density for non-prevalent immunohistochemical labelling (i.e. by T1, $G\alpha_{s/olf}$; T3, $G\alpha_{q/11/14}$; T4, $G\alpha_{i-1/2/3}$; T5, $G\alpha_{i-3}$), allowing for manual counting.

```
// Author: Andrew Mehnert
// History: 21/11/2018 - Initial version
//          18/1/2019 - Made change to fix function renaming introduced in
MorphoLibJ: replaced run("Region Morphometry") with run("Analyze Regions",
"area");

run("Close All");
// Ask the user for the file to be processed
filepathname = File.openDialog("Select the file to be processed");

setBatchMode(true);
// Load in the file
run("Bio-Formats", "open=["+filepathname+"] autoscale color_mode=Default
open_all_series rois_import=[ROI manager] view=Hyperstack stack_order=XYCZT
contains=[] name=["+filepathname+"]");

// Get unit of length (as a string) and the pixel dimensions
getVoxelSize(pixelWidth, pixelHeight, pixelDepth, unit);
print("Pixel width "+pixelWidth+" "+unit);
print("Pixel height "+pixelHeight+" "+unit);

// Validate voxel size
//run("Properties...");

// Keep only the first two channels
rename("source");
run("Make Substack...", "channels=1-2");
close("source");
rename("source");

// Perform maximum intensity projection on each channel
run("Z Project...", "projection=[Max Intensity]");
close("source");

// Rotate 90 degrees to span monitor screen width and reinstate units and pixel
size
run("Rotate 90 Degrees Left");
setVoxelSize(pixelWidth, pixelHeight, pixelDepth, unit);

// Split the channels and enhance the contrast in each
run("Split Channels");
selectWindow("C1-MAX_source");
run("Enhance Contrast...", "saturated=0.3 normalize equalize");
selectWindow("C2-MAX_source");
run("Enhance Contrast...", "saturated=0.3 normalize equalize");
```

```
// Apply Frangi's vesselness filter to the second channel
run("Tubeness", "sigma=1.0000 use");
run("Enhance Contrast...", "saturated=0.3 normalize");
run("16-bit");

// Merge all channels together again with C4 equal to the vesselness response
run("Merge Channels...", "c1=C2-MAX_source c3=C1-MAX_source c4=[tubeness of C2-
MAX_source] create");
setBatchMode(false);

// Ask user to roughly outline the tissue and then clear everything outside this
ROI
run("Channels Tool...");
setTool("freehand");
waitForUser("Please use the freehand tool to roughly outline the tissue.\nPress
OK when you are done.");
setBackgroundColor(0, 0, 0);
run("Clear Outside");

// Now create the tissue mask from channel 1
run("Duplicate...", "title=TissueMask duplicate channels=1");     // Duplicate
the channel
run("Remove Outliers...", "radius=30 threshold=0 which=Bright");  // Denoise
run("Threshold...");                                              // Run the
theshold tool
waitForUser("Use the threshold tool to select the tissue of interest.\nNOTE:
Ensure that \'Dark background\' is ticked and click \'Apply\' to apply the
threshold.\n\nPress OK when you are done.");

// Ask the user to place one or more points on the connected components to be
retained
setTool("multipoint");
waitForUser("Please place a point on each section of tissue you want to
retain.\nPress OK when you are done.");
run("Interactive Morphological Reconstruction", "type=[By Dilation]
connectivity=4");
close("TissueMask");

// Measure the area of the selected tissue
//run("Region Morphometry"); old version of MorphoLibJ
run("Analyze Regions", "area");
selectWindow("TissueMask-rec-Morphometry");
area = Table.get("Area",0);
close("TissueMask-rec-Morphometry");

// Allow the user to select the objects of interest
Table.create("Density table");
selectWindow("Composite");
run("Select None");
setTool("multipoint");
waitForUser("Please use the multipoint tool to select the objects of
interest.\nPress Ok when you are done");

// Store the area and object count values in the Density table
getSelectionCoordinates(x, y);
selectWindow("Density table");
```

```
Table.set("TissueArea", 0, area);
Table.set("NumObjects", 0, x.length);
Table.update;

// Flatten the composite image and selected points to a single RGB image
selectWindow("Composite");
run("Add Selection...");
run("Flatten");
run("Tile");
```

Supplementary Material S2. Script developed and used in Fiji (ImageJ 1.52n, NIH, USA) to assess cell density for prevalent immunohistochemical labelling (i.e. by T2, $G\alpha_o$) for prevalent labelling, not allowing for manual counting, thus using the amount of labelling per unit area as a proxy for cell density.

```
// Author: Andrew Mehnert
// History: 17/1/2019 - Initial version

roiManager("reset");
close("ROI Manager");
run("Clear Results");
run("Close All");

// Ask the user for the file to be processed
filepathname = File.openDialog("Select the file to be processed");
filepath = File.directory;
print(filepath);
filename = File.nameWithoutExtension;
print(filename);

setBatchMode(true);
// Load in the file
run("Bio-Formats", "open=["+filepathname+"] autoscale color_mode=Default
open_all_series rois_import=[ROI manager] view=Hyperstack stack_order=XYCZT
contains=[] name=["+filepathname+"]");

// Get unit of length (as a string) and the pixel dimensions
getVoxelSize(pixelWidth, pixelHeight, pixelDepth, unit);
print("Pixel width "+pixelWidth+" "+unit);
print("Pixel height "+pixelHeight+" "+unit);

// Validate voxel size
//run("Properties...");

// Keep only the first two channels
rename("source");
run("Make Substack...", "channels=1-2");
close("source");
rename("source");

// Perform maximum intensity projection on each channel
run("Z Project...", "projection=[Max Intensity]");
close("source");

// Rotate 90 degrees to span monitor screen width and reinstate units and pixel
size
run("Rotate 90 Degrees Left");
setVoxelSize(pixelWidth, pixelHeight, pixelDepth, unit);

// Split the channels and enhance the contrast in each
run("Split Channels");
selectWindow("C1-MAX_source");
run("Enhance Contrast...", "saturated=0.3 normalize equalize");
selectWindow("C2-MAX_source");
run("Enhance Contrast...", "saturated=0.3 normalize equalize");
```

```
// Apply Frangi's vesselness filter to the second channel
run("Tubeness", "sigma=1.0000 use");
run("Enhance Contrast...", "saturated=0.3 normalize");
run("16-bit");

// Merge all channels together again with C4 equal to the vesselness response
run("Merge Channels...", "c1=C2-MAX_source c3=C1-MAX_source c4=[tubeness of C2-
MAX_source] create");
rename("source");
setBatchMode(false);
filepathname = filepath+filename+"_source.tif"; // Save the tissue mask
saveAs("Tiff", filepathname);


// Ask user to roughly outline the tissue and then clear everything outside this
ROI
run("Channels Tool...");
setTool("freehand");
waitForUser("Please use the freehand tool to roughly outline the tissue.\nPress
OK when you are done.");
setBackgroundColor(0, 0, 0);
run("Clear Outside");

// Now create the tissue mask from channel 1
run("Duplicate...", "title=TissueMask duplicate channels=1");     // Duplicate
the channel
run("Remove Outliers...", "radius=30 threshold=0 which=Bright");  // Denoise
run("Threshold...");                                              // Run the
theshold tool
waitForUser("Use the threshold tool to select the tissue of interest.\nNOTE:
Ensure that \'Dark background\' is ticked and click \'Apply\' to apply the
threshold.\n\nPress OK when you are done.");

// Ask the user to place one or more points on the connected components to be
retained
setTool("multipoint");
waitForUser("Please place a point on each section of tissue you want to
retain.\nPress OK when you are done.");
run("Interactive Morphological Reconstruction", "type=[By Dilation]
connectivity=4");
close("TissueMask");
close("Threshold");

// Measure the area of the selected tissue
//run("Region Morphometry"); old version of MorphoLibJ
run("Analyze Regions", "area");
selectWindow("TissueMask-rec-Morphometry");
tissueArea = Table.get("Area",0);
close("TissueMask-rec-Morphometry");
print("Mask area is "+tissueArea+" square microns");
run("Clear Results");
selectWindow("TissueMask-rec");
filepathname = filepath+filename+"_tissue_mask.tif"; // Save the tissue mask
saveAs("Tiff", filepathname);
close();

// Allow the user to select ROIs of interest
selectWindow("source");
run("Select None");
```

```
run("ROI Manager...");
roiManager("Show All");
roiManager("Show All with labels");
setTool("polygon");
waitForUser("Please use the polygon tool to select a region of interest and add
it to the ROI manager [t].\nPress Ok when you have finished adding ROIs");

filepathname = filepath+filename+"_RoiSet.zip"; // Save the ROIs
roiManager("Save", filepathname);

// Loop through each ROI and have the user verify number of nuclei
Table.create("Density table");
n = roiManager("count");
for (i=0; i<n; i++) {
//      setBatchMode(true);
        selectWindow("source");
        roiManager("deselect")
    roiManager("Select", i);
        run("Duplicate...", "duplicate ");
        run("Split Channels");
    selectWindow("C1-source-1"); // This is the red-stained image
    run("Restore Selection");
    getStatistics(area, mean);
    selectWindow("Density table");
    Table.set("ROI", i , i+1);
    Table.set("area", i, area);
    Table.set("stain mean", i, mean);
    Table.update;
    selectWindow("C1-source-1"); // This is the red-stained image
    run("Morphological Filters", "operation=[White Top Hat] element=Disk
radius=2"); //White tophat of red-stained image
    close("C1-source-1");
    run("Restore Selection");
    getStatistics(area, mean);
    selectWindow("Density table");
    Table.set("stain mean tophat", i, mean);
    Table.update;
    close("C1-source-1-White Top Hat");

        selectWindow("C3-source-1"); // This is the tubeness image
    run("Restore Selection");
    getStatistics(area, mean);
    selectWindow("Density table");
    Table.set("stain mean tubeness", i, mean);
    Table.update;
    close("C3-source-1");
    setBatchMode(false);

    selectWindow("C2-source-1"); // This is the blue-stained image
    run("Restore Selection");
    run("In [+]");
    run("In [+]");
    run("In [+]");
    run("In [+]");
    run("Clear Outside");
    if (i==0){
```

```
      run("Find Maxima...", "noise=5000 output=Count exclude"); // This statement
serves only to set initial values;
      run("Clear Results");
    }
    run("Find Maxima...");
    close("C2-source-1");
}
selectWindow("Results");
counts = Table.getColumn("Count");
selectWindow("Density table");
Table.setColumn("ROI nucleus count",counts);
Table.update;
Table.set("tissue area", 0, tissueArea);
Table.update;
filepathname = filepath+filename+"_results.csv";
saveAs("Results", filepathname); // Save the Density Table
close("Results");
```

Supplementary Material S3. Comparison of un-morphed (left) and morphed (right) features of sample the specimen of *Chiloscyllium punctatum* used (CP1). The curvature in the barbels has been corrected along with the flattening of the nostril on the left-hand side of CP1.

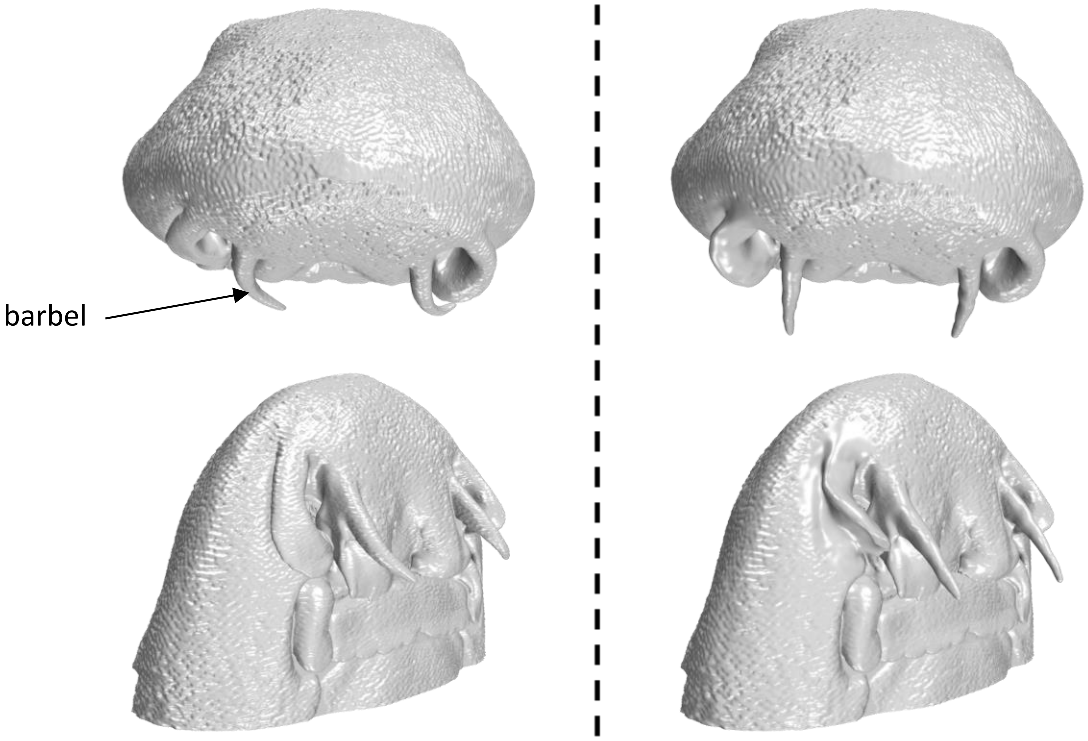

Supplementary Material S4. Meshing parameters used to produce a course, medium and fine density mesh for CP1. A target scaling relationship between meshes of $2^{1/3}$ was applied ($r = 2^{1/3}$).

**Mesh Property**	**Coarse Mesh**	**Medium Mesh**	**Fine Mesh**
Polyhedral Density	0.5	1	2
Number of Prism Layers	6	12	24
Prism Layer Stretching	1.149	1.065	1.031
Number of Cells	531 580	828 787	1 394 525

Supplementary Material S5. Results of grid convergence index (GCI) for surface averaged WSS (SAWSS) across each half of the left and right rosettes of CP1. GCI was calculated using the non-uniform refinement ratio formulation.

Parameter of Interest	Coarse Mesh	Medium Mesh	Fine Mesh	GCI
SAWSS across LR1 (Pa)	1.855	1.715	1.778	2.53%
SAWSS across LR2 (Pa)	0.467	0.440	0.427	1.78%
SAWSS across RR1 (Pa)	1.177	1.069	1.106	1.41%
SAWSS across RR2 (Pa)	0.355	0.321	0.315	0.23%

Supplementary Material S6 – Video 1. Animation showing the lateral segregation existing in the olfactory bulb olfactory bulb of *Chiloscyllium punctatum*, by revealing the segmented clusters of glomeruli. Created and edited in Avizo (v9.2.0). From Camilieri-Asch (2019).

Supplementary Material S7. Model fitted mean values generated by *post-hoc* Tukey tests for the density of non-prevalent labelled Olfactory Receptor Neuron cells in the olfactory mucosa of *Chiloscyllium punctatum* (*n*=2). Error bars indicate standard errors. CP, *Chiloscyllium punctatum* (specimens 7 and 8); T; treatment (marker used); T1, G$\alpha_{s/olf}$; T3, Gα_q; T4, G$\alpha_{i-1/2/3}$; T5, Gα_{i-3}; L1-2, medial lamellae pairs; L3-4, lateral lamellae pairs. See figure 5.2 for reference to positions of lamellae pairs sampled.

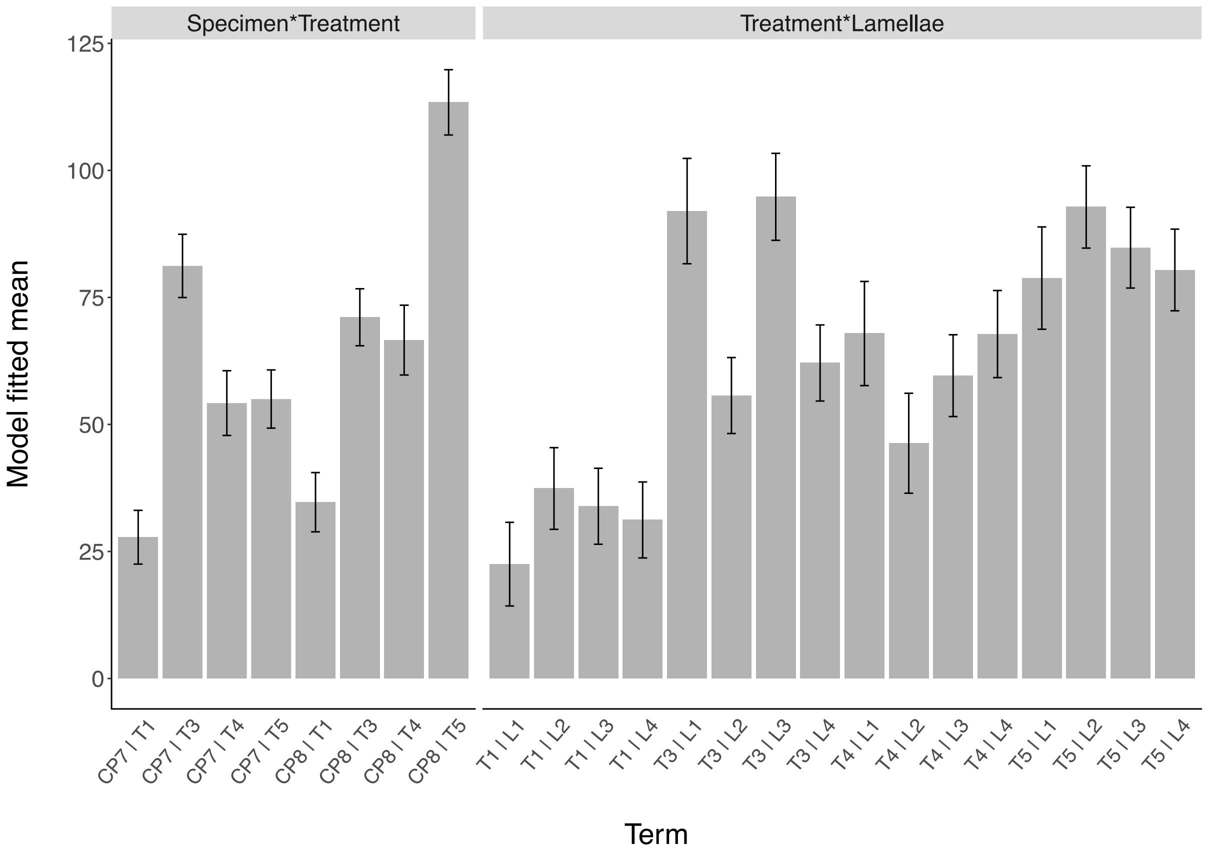

Supplementary Material S8. Outputs of *post-hoc* Tukey tests for significant interacting terms tested in the linear regression model for the density of non-prevalent labelled Olfactory Receptor Neuron cells in the olfactory mucosa of *Chiloscyllium punctatum* (*n*=2). Highlighted rows indicate statistical results of interest; **(1)** biologically-relevant contrast terms; SE, standard error; T1, G$\alpha_{s/olf}$; T3, G$\alpha_{q/11/14}$; T4, G$\alpha_{i-1/2/3}$; T5, Gα_{i-3}.

Interaction tested	contrast term (1)	estimate	SE	p-value (adjusted)
Specimen*Treatment	CP7,T1 – CP8,T1	-6.90	7.87	0.3824
	CP7,T1 – CP7,T3	-53.38	8.16	< 0.0001***
	CP7,T1 – CP7,T4	-26.40	8.28	0.0019**
	CP7,T1 – CP7,T5	-27.22	7.80	0.0007**
	CP8,T1 – CP8,T3	-36.44	7.89	< 0.0001***
	CP8,T1 – CP8,T4	-31.92	8.62	0.0003**
	CP8,T1 – CP8,T5	-78.68	8.24	< 0.0001***
	CP7,T3 – CP8,T3	10.05	8.14	0.2196
	CP7,T3 – CP7,T4	26.98	8.67	0.0024**
	CP7,T3 – CP7,T5	26.16	8.25	0.002**
	CP8,T3 – CP8,T4	4.51	8.62	0.6017
	CP8,T3 – CP8,T5	-42.25	8.24	< 0.0001***
	CP7,T4 – CP8,T4	-12.43	8.66	0.1542
	CP7,T4 – CP7,T5	-0.82	8.39	0.9223
	CP8,T4 – CP8,T5	-46.76	8.80	< 0.0001***
	CP7,T5 – CP8,T5	-58.36	8.31	< 0.0001***
Treatment*Lamellae	T1,L1 - T3,L1	-69.56	12.40	< 0.0001***
	T1,L1 - T4,L1	-45.47	12.20	0.0003**
	T1,L1 - T5,L1	-56.35	12.00	< 0.0001***
	T1,L1 - T1,L2	-14.95	11.40	0.1910
	T1,L1 - T1,L3	-11.47	11.10	0.3046
	T1,L1 - T1,L4	-8.74	11.10	0.4334
	T3,L1 - T4,L1	24.09	12.70	0.0605
	T3,L1 - T5,L1	13.20	12.50	0.2934
	T3,L1 - T3,L2	36.27	12.80	0.0054**
	T3,L1 - T3,L3	-2.80	13.30	0.8339
	T3,L1 - T3,L4	29.96	12.80	0.0209*
	T4,L1 - T5,L1	-10.88	12.30	0.3779
	T4,L1 - T4,L2	21.63	13.30	0.1056
	T4,L1 - T4,L3	8.34	12.70	0.5139
	T4,L1 - T4,L4	0.10	13.00	0.9940
	T5,L1 - T5,L2	-13.99	12.50	0.2666
	T5,L1 - T5,L3	-6.00	12.90	0.6419
	T5,L1 - T5,L4	-1.55	12.50	0.9012
	T1,L2 - T3,L2	-18.34	11.00	0.0976
	T1,L2 - T4,L2	-8.89	12.40	0.4738
	T1,L2 - T5,L2	-55.40	11.20	< 0.0001***
	T1,L2 - T1,L3	3.47	11.00	0.7524
	T1,L2 - T1,L4	6.20	11.00	0.5733

T3,L2 - T4,L2	9.45	12.40	0.4463
T3,L2 - T5,L2	-37.06	11.00	0.0011**
T3,L2 - T3,L3	-39.07	11.40	0.0008**
T3,L2 - T3,L4	-6.31	10.60	0.5518
T4,L2 - T5,L2	-46.51	12.40	0.0003**
T4,L2 - T4,L3	-13.29	12.40	0.2865
T4,L2 - T4,L4	-21.53	12.70	0.0921
T5,L2 - T5,L3	7.99	11.40	0.4835
T5,L2 - T5,L4	12.44	11.20	0.2674
T1,L3 - T3,L3	-60.88	11.40	< 0.0001***
T1,L3 - T4,L3	-25.65	11.00	0.0214*
T1,L3 - T5,L3	-50.88	10.90	< 0.0001***
T1,L3 - T1,L4	2.73	10.60	0.7969
T3,L3 - T4,L3	35.23	11.60	0.003**
T3,L3 - T5,L3	10.00	11.60	0.3889
T3,L3 - T3,L4	32.75	11.40	0.0048**
T4,L3 - T5,L3	-25.23	11.30	0.0277*
T4,L3 - T4,L4	-8.25	11.70	0.4833
T5,L3 - T5,L4	4.45	11.30	0.6957
T1,L4 - T3,L4	-30.85	10.60	0.0043**
T1,L4 - T4,L4	-36.63	11.40	0.0017**
T1,L4 - T5,L4	-49.16	11.00	< 0.0001***
T3,L4 - T4,L4	-5.77	11.40	0.6127
T3,L4 - T5,L4	-18.31	11.00	0.0983
T4,L4 - T5,L4	-12.54	11.60	0.2814

Supplementary Material S9. Model fitted mean values (.10^6) generated by *post-hoc* Tukey tests for the density of prevalent Olfactory Receptor Neuron cells labelled by the marker $G\alpha_o$ in the olfactory mucosa of *Chiloscyllium punctatum* (CP, *n*=2). Error bars indicate standard errors. CP7, specimen 7; CP8, specimen 8; L1-2, medial lamellae pairs; L3-4, lateral lamellae pairs; s1-6, section positions on the lamellae, from anterior to posterior. See figure 2 for reference to positions of both lamellae pairs sampled and sections on lamellae.

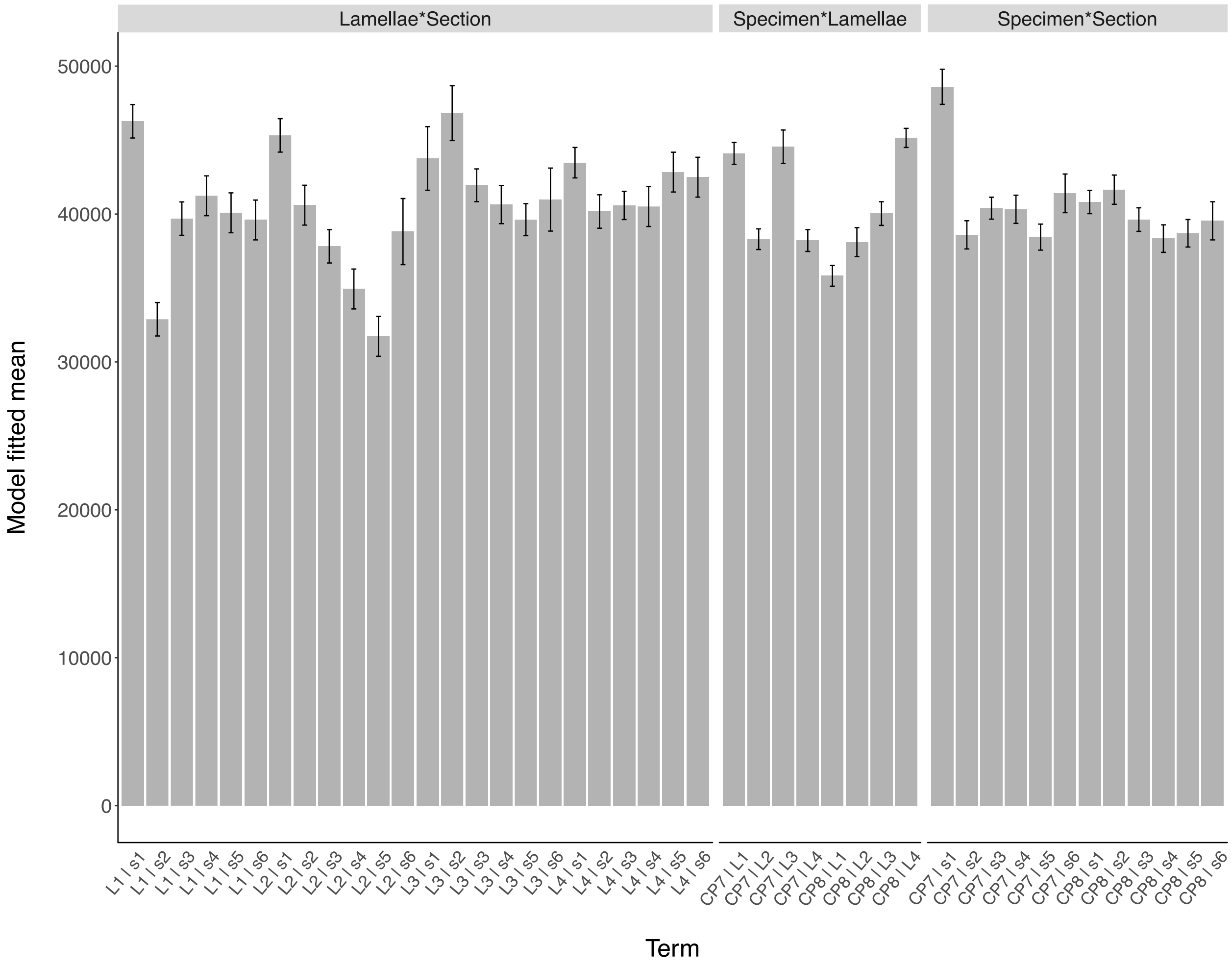

Supplementary Material S10. Outputs of *post-hoc* Tukey tests for significant interacting terms tested in the linear regression model for the density of prevalent labelled Olfactory Receptor Neuron cells in the olfactory mucosa of *Chiloscyllium punctatum* (*n*=2). Highlighted rows indicate statistical results of interest; [(1)] biologically-relevant terms; SE, standard error.

Interaction tested	contrast term [(1)]	estimate	SE	p-value (adjusted)
Lamellae*Section	L1,s1 - L1,s2	13382.2	1587	<.0001***
	L1,s1 - L1,s3	6579	1609	0.0001**
	L1,s1 - L1,s4	5035.7	1758	0.0045**
	L1,s1 - L1,s5	6184.9	1758	0.0005**
	L1,s1 - L1,s6	6674.2	1758	0.0002**
	L2,s1 - L2,s2	4720.5	1759	0.0078**
	L2,s1 - L2,s3	7499.6	1582	<.0001***
	L2,s1 - L2,s4	10384.9	1759	<.0001***
	L2,s1 - L2,s5	13587.8	1759	<.0001***
	L2,s1 - L2,s6	6507.5	2463	0.0088**
	L3,s1 - L3,s2	-3063.6	2962	0.302
	L3,s1 - L3,s3	1814.1	2351	0.4411
	L3,s1 - L3,s4	3119.1	2493	0.212
	L3,s1 - L3,s5	4138.2	2450	0.0924
	L3,s1 - L3,s6	2779.7	2835	0.3278
	L4,s1 - L4,s2	3301.6	1510	0.0297*
	L4,s1 - L4,s3	2891.2	1401	0.04*
	L4,s1 - L4,s4	2966	1694	0.0811
	L4,s1 - L4,s5	638.1	1694	0.7067
	L4,s1 - L4,s6	982.2	1694	0.5625
	L1,s2 - L1,s3	-6803.1	1609	<.0001***
	L1,s2 - L1,s4	-8346.5	1758	<.0001***
	L1,s2 - L1,s5	-7197.3	1758	0.0001**
	L1,s2 - L1,s6	-6708	1758	0.0002**
	L2,s2 - L2,s3	2779.2	1756	0.1148
	L2,s2 - L2,s4	5664.4	1905	0.0032**
	L2,s2 - L2,s5	8867.3	1905	<.0001***
	L2,s2 - L2,s6	1787	2610	0.4941
	L3,s2 - L3,s3	4877.7	2206	0.0279*
	L3,s2 - L3,s4	6182.6	2267	0.0068**
	L3,s2 - L3,s5	7201.8	2120	0.0008**
	L3,s2 - L3,s6	5843.3	2948	0.0486
	L4,s2 - L4,s3	-410.4	1478	0.7815
	L4,s2 - L4,s4	-335.6	1758	0.8488

	L4,s2 - L4,s5	-2663.5	1758	0.131
	L4,s2 - L4,s6	-2319.4	1758	0.1882
	L1,s3 - L1,s4	-1543.3	1755	0.3801
	L1,s3 - L1,s5	-394.1	1755	0.8225
	L1,s3 - L1,s6	95.2	1755	0.9568
	L2,s3 - L2,s4	2885.3	1756	0.1017
	L2,s3 - L2,s5	6088.1	1756	0.0006**
	L2,s3 - L2,s6	-992.1	2460	0.687
	L3,s3 - L3,s4	1304.9	1690	0.4408
	L3,s3 - L3,s5	2324.1	1566	0.1391
	L3,s3 - L3,s6	965.6	2332	0.6792
	L4,s3 - L4,s4	74.8	1649	0.9639
	L4,s3 - L4,s5	-2253.1	1649	0.1731
	L4,s3 - L4,s6	-1909	1649	0.2482
	L1,s4 - L1,s5	1149.2	1905	0.5468
	L1,s4 - L1,s6	1638.5	1905	0.3904
	L2,s4 - L2,s5	3202.9	1905	0.0939
	L2,s4 - L2,s6	-3877.4	2610	0.1386
	L3,s4 - L3,s5	1019.2	1686	0.546
	L3,s4 - L3,s6	-339.3	2475	0.8911
	L4,s4 - L4,s5	-2327.9	1905	0.2227
	L4,s4 - L4,s6	-1983.8	1905	0.2986
	L1,s5 - L1,s6	489.3	1905	0.7975
	L2,s5 - L2,s6	-7080.3	2610	0.0071**
	L3,s5 - L3,s6	-1358.5	2431	0.5769
	L4,s5 - L4,s6	344.1	1905	0.8568
Specimen*Lamellae	CP7,L1 – CP8,L1	8279.5	1021	<.0001***
	CP7,L1 – CP7,L2	5801.5	1015	<.0001***
	CP7,L1 – CP7,L3	-449.9	1342	0.7376
	CP7,L1 – CP7,L4	5891.5	1038	<.0001***
	CP8,L1 – CP8,L2	-2283.4	1194	0.057
	CP8,L1 – CP8,L3	-4212.9	1072	0.0001**
	CP8,L1 – CP8,L4	-9323.1	946	<.0001***
	CP7,L2 – CP8,L2	194.6	1184	0.8696
	CP7,L2 – CP7,L3	-6251.5	1341	<.0001***
	CP7,L2 – CP7,L4	89.9	1014	0.9294
	CP8,L2 – CP8,L3	-1929.4	1263	0.1279
	CP8,L2 – CP8,L4	-7039.7	1156	<.0001***
	CP7,L3 – CP8,L3	4516.6	1407	0.0015**
	CP7,L3 – CP7,L4	6341.4	1338	<.0001***
	CP8,L3 – CP8,L4	-5110.3	1032	<.0001***

	CP7,L4 – CP8,L4	-6935.1	973	<.0001***
Specimen*Section	CP10,s1 - CP11,s1	7793.1	1373	<.0001***
	CP10,s1 - CP10,s2	10007.6	1521	<.0001***
	CP10,s1 - CP10,s3	8207.1	1370	<.0001***
	CP10,s1 - CP10,s4	8283	1521	<.0001***
	CP10,s1 - CP10,s5	10165.4	1501	<.0001***
	CP10,s1 - CP10,s6	7205.2	1686	<.0001***
	CP11,s1 - CP11,s2	-837.3	1255	0.5054
	CP11,s1 - CP11,s3	1184.9	1112	0.2879
	CP11,s1 - CP11,s4	2469.8	1217	0.0434*
	CP11,s1 - CP11,s5	2109.1	1217	0.0842
	CP11,s1 - CP11,s6	1266.6	1489	0.3957
	CP10,s2 - CP11,s2	-3051.7	1372	0.027*
	CP10,s2 - CP10,s3	-1800.5	1206	0.1366
	CP10,s2 - CP10,s4	-1724.5	1347	0.2015
	CP10,s2 - CP10,s5	157.8	1296	0.9032
	CP10,s2 - CP10,s6	-2802.4	1616	0.0841
	CP11,s2 - CP11,s3	2022.1	1280	0.1155
	CP11,s2 - CP11,s4	3307	1362	0.0159*
	CP11,s2 - CP11,s5	2946.4	1363	0.0316*
	CP11,s2 - CP11,s6	2103.9	1610	0.1925
	CP10,s3 - CP11,s3	770.9	1100	0.4841
	CP10,s3 - CP10,s4	75.9	1206	0.9498
	CP10,s3 - CP10,s5	1958.3	1157	0.0919
	CP10,s3 - CP10,s6	-1001.9	1474	0.4974
	CP11,s3 - CP11,s4	1284.9	1225	0.295
	CP11,s3 - CP11,s5	924.2	1224	0.4509
	CP11,s3 - CP11,s6	81.7	1507	0.9568
	CP10,s4 - CP11,s4	1979.9	1332	0.1384
	CP10,s4 - CP10,s5	1882.4	1296	0.1478
	CP10,s4 - CP10,s6	-1077.8	1616	0.5053
	CP11,s4 - CP11,s5	-360.7	1316	0.7843
	CP11,s4 - CP11,s6	-1203.2	1591	0.4502
	CP10,s5 - CP11,s5	-263.2	1276	0.8368
	CP10,s5 - CP10,s6	-2960.2	1597	0.0649
	CP11,s5 - CP11,s6	-842.5	1591	0.5969
	CP10,s6 - CP11,s6	1854.5	1992	0.3529

Supplementary Material S11 – Video 2. Rotational animation of the flow velocity streamlines in the olfactory cavities of *Chiloscyllium punctatum* at the highest flow velocity speed simulated (120cm.s^{-1}). Created and exported from figures in STAR-CCM+ (v12.06, Siemens, Berlin).
